# Bioinspired Multiscale Wrinkling Patterns on Curved Substrates: An Overview

**DOI:** 10.1007/s40820-020-00436-y

**Published:** 2020-04-25

**Authors:** Yinlong Tan, Biru Hu, Jia Song, Zengyong Chu, Wenjian Wu

**Affiliations:** College of Liberal Arts and Science, National University of Defense Technology, Changsha, 410073 People’s Republic of China

**Keywords:** Surface instability, Wrinkling patterns, Substrate curvature, Micro/nano fabrications, Low-dimensional materials

## Abstract

The surface wrinkling of biological tissues is ubiquitous in nature. Accumulating evidence suggests that the mechanical force plays a significant role in shaping the biological morphologies. Controlled wrinkling has been demonstrated to be able to spontaneously form rich multiscale patterns, on either planar or curved surfaces. The surface wrinkling on planar substrates has been investigated thoroughly during the past decades. However, most wrinkling morphologies in nature are based on the curved biological surfaces and the research of controllable patterning on curved substrates still remains weak. The study of wrinkling on curved substrates is critical for understanding the biological growth, developing three-dimensional (3D) or four-dimensional (4D) fabrication techniques, and creating novel topographic patterns. In this review, fundamental wrinkling mechanics and recent advances in both fabrications and applications of the wrinkling patterns on curved substrates are summarized. The mechanics behind the wrinkles is compared between the planar and the curved cases. Beyond the film thickness, modulus ratio, and mismatch strain, the substrate curvature is one more significant parameter controlling the surface wrinkling. Curved substrates can be both solid and hollow with various 3D geometries across multiple length scales. Up to date, the wrinkling morphologies on solid/hollow core–shell spheres and cylinders have been simulated and selectively produced. Emerging applications of the curved topographic patterns have been found in smart wetting surfaces, cell culture interfaces, healthcare materials, and actuators, which may accelerate the development of artificial organs, stimuli-responsive devices, and micro/nano fabrications with higher dimensions.

## Highlights

- An overview of the formation mechanisms, fabrication methods, and applications of bioinspired wrinkling patterns on curved substrates is provided.
- The effect of substrate curvature is described in detail to clarify the difference of wrinkling patterns between planar and curved substrates.
- Opportunities and challenges of the surface wrinkling in the biofabrication, three-dimensional micro/nano fabrication, and four-dimensional printing are discussed.

## Graphical abstract

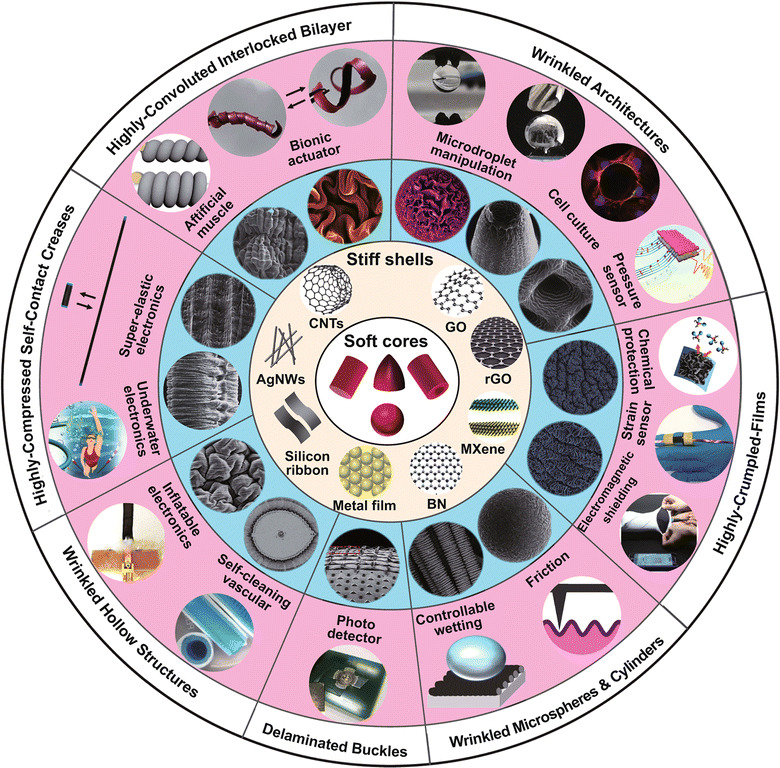

## Introduction

**Fig. 1:**
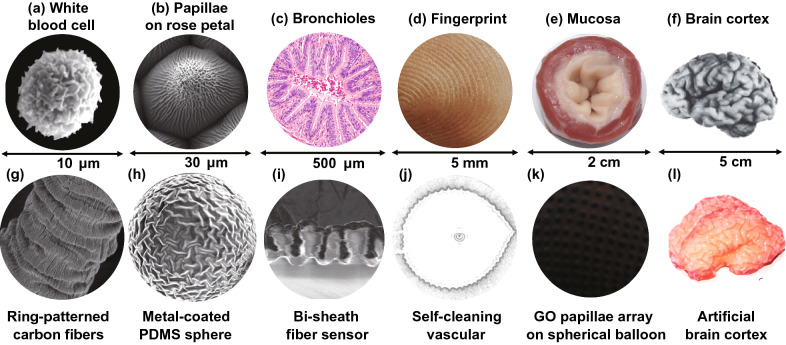
Wrinkling patterns on curved substrates **a**–**f** from nature and **g**–**l** in labs across multiple length scales. **a** White blood cell. The extremely wrinkled membrane enables the cell to expand and contract under chemotaxis and phagocytosis [3]. Copyright 2008 Springer Nature. **b** Papillae on the rose petal. **c** Cross-sectional view of the bronchioles [15]. Copyright 2018 Elsevier. **d** Photograph of fingerprint. **e** Mucosa of bovine esophagus [17]. Copyright 2011 Elsevier. **f** Human brain [11]. Copyright 2010 Elsevier. **g** Ring-patterned carbon fibers [58]. Copyright 2019 Elsevier. **h** Metal-coated polydimethylsiloxane (PDMS) spheres for tunable frication [59]. Copyright 2019 Wiley. **i** Bi-sheath fiber for strain sensor [60]. Copyright 2017 Wiley. **j** Wrinkled tube for anti-thrombotic [63]. Copyright 2019 Elsevier. **k** Papillae array for microdroplet manipulation [54]. Copyright 2019 Wiley. **l** Swelling-induced artificial brain cortex with sulci and gyri [65]. Copyright 2016 Springer Nature

Wrinkling morphologies are ubiquitous on curved biological surfaces across multiple length scales such as spheroidal cells, cylindrical fingers, and tubular mucosa (Fig. 1). In many situations, dynamic wrinkling morphologies are capable of regulating the physiological, biochemical, and physical properties of biological surfaces [1–18]. For example, the wrinkled cell membrane enables large surface area and enhanced deformability [1–3], the sulci and gyri of brain cortex greatly increase intellectual capacity [7–10], the hierarchical villi of small intestine enhances nutrients absorption [12–14], and the papillae array on the rose petal enables both superhydrophobicity and high adhesion to water [18]. These functional wrinkled structures on biological surfaces have fascinated and challenged scientists and engineers for decades [1–10, 12–14, 16, 17, 19, 20]. Although the biological morphologies are significantly influenced by biochemical and genetic factors [19], accumulating evidence suggests that the mechanical force plays an important role in shaping the morphologies of the biological tissues [2–6, 9, 10, 14, 17, 20]. Sharon et al. [20] demonstrated that a flat eggplant leaf could buckle into a wavy one under non-uniform stress induced by the localized expansion of the leaf edge. By establishing a mechanics model of a spheroidal core–shell system, Yin et al. [21] simulated a series of buckling morphologies that are quite similar to the morphogenesis of some plant tissues. Their analysis indicated that mechanical-driven self-assembly has important implications on shaping the biological morphologies. By coating a soft shell on an elastic hemispherical core, and then swelling the obtained core–shell hemisphere in organic solvents, the formation of the highly convoluted pattern consisting of cusped sulci and smooth gyri can be induced by the swelling-induced tangential stress [22]. Similarly, the mechanical stress that triggers the wrinkling of biological surfaces can also be induced by the growing biological tissues.

Besides wrinkling patterns in nature, diverse functional micro/nano patterns can be obtained by traditional engineering approaches including photolithography and ion etching. However, the photolithography-based techniques are effective only to a few photoresists on planar surfaces, and the ion-etching method is extremely expensive and inefficient, which limits their applications in large-area micro/nano fabrications [23–25]. Moreover, the fabrication of high-aspect-ratio micro/nanostructures on non-planar surfaces via these techniques is still a challenge. Surface wrinkling of stiff coatings in layered systems are traditionally regarded as defects or failures in engineering [26–28]. However, these engineering defects in layered systems also provide inspirations for developing low-cost and highly efficient patterning techniques in material science and mechanical engineering [29–35]. In 1998, Bowden et al. [36] demonstrated that microwrinkles could be obtained by simply cooling a metal-coated polymer substrate, suggesting that the mechanical self-assembly could be a facile approach for micro and nano fabrications. In the past decades, the mechanical self-assembly of thin films in layered systems has been well developed for low-cost and highly efficient fabrications of micro/nanostructures using diverse film materials, such as inorganics [32, 37], metals [36, 38], polymers [30, 39], and novel carbon materials [33, 35]. Besides, the mechanical force that drives the assembly of films can be induced by chemical reactions (e.g., surface polymerization and surface oxidation) [30], or physical stimuli (e.g., thermal shrinking, prestrain releasing, and electronic actuation) [32]. Moreover, rich parameters can be used to manipulate the wrinkling of thin films on soft substrates, such as material properties of the film and the substrate, mismatch strain, and stress direction, enabling unprecedented control over the morphology and feature size of the patterns and structures for diverse applications such as flexible electronics [33, 40], cell culture interfaces [41, 42], reversible patterning [43–45], and super-wetting surfaces [46–48].

Beyond planar substrates, more and more curved substrates such as spheres, cylinders, cones, and tubes are harnessed to fabricate and simulate wrinkling patterns that are similar to the patterns on biological surfaces [49, 50]. By depositing SiO_2_ shells on Ag microspheres, Li et al. [51] reproduced Fibonacci number patterns by cooling the core–shell spheres. With adhering thin polyvinyl chloride films on polyurethane cylinders, Yin et al. [52] obtained a set of gears by dehydration-induced circumferential compressive stress. Liu et al. [53] fabricated hierarchical buckles on a superelastic fiber by releasing a prestretched fiber wrapped with carbon nanotube sheets. With coating graphene oxide films on inflated latex balloons, Tan et al. [54, 55] constructed a variety of 3D wrinkled structures on hollow substrates by deflation. Rich parameters including substrate curvature, shell thickness, modulus ratio, and mismatch strain were demonstrated to be able to control the wrinkling of thin shells on curved substrates, enabling 3D fabrication of micro and nano structures for various applications such as photo detectors [56, 57], controllable adhesion [54, 58, 59], superelastic electronics [53, 60], chemical barriers [61], electromagnetic shielding [62], inflatable devices [63, 64], bionic cortical structures [65], and strong actuators [53, 55] (Fig. 1). A number of reviews concentrated on mechanics, morphogenesis, and applications of surface instability in soft materials have been reported [29–35, 49, 50]. For example, Rodríguez-Hernández [30] systematically summarized the fabrication approaches and application fields of diverse wrinkling patterns on polymer substrates. Wang and Zhao [31] presented a brief overview about multimodal surface instabilities for multifunctional patterning. Chen et al*.* [35] provided a review on higher dimensional patterning with two-dimensional materials by mechanical assembly. Recently, Hu et al. [33] summarized the fabrication methods for conductive buckled structures and their applications in wearable electronics. However, these reviews mainly focused on the fabrications and applications of wrinkling patterns on planar substrates and rare reviews concentrated on the research progress in surface instability of curved core–shell systems. Therefore, a systematic overview about the formation mechanisms, fabrication techniques, and applications of wrinkling patterns on curved substrates is needed. It is crucial for understanding and mimicking surface instability on curved substrates, developing template-free 3D fabrication methods, and discovering novel patterns for new applications.

Because most previous studies mainly focused on core–shell systems, the surface instability of closed core–shell structures will be the center of this review. Mechanics behind the surface wrinkling on the planar and curved substrates is introduced firstly. Then recent advances in the experimental fabrication of wrinkling patterns in the typical curved core–shell systems are provided. Emerging applications of some representative patterns are summarized according to the geometries and materials of the cores and the shells. Current challenges and future perspectives of the wrinkling patterns on curved substrates are finally discussed.

## Mechanics Behind the Wrinkles

### Planar Substrates

**Fig. 2:**
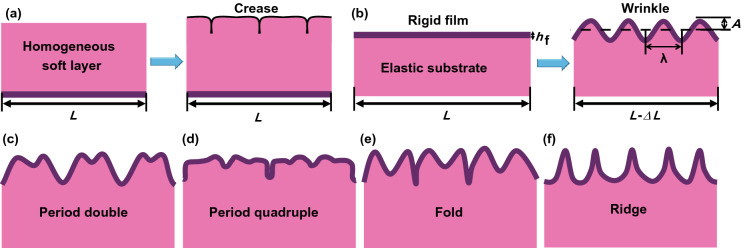
**a** Schematic of crease formation on the surface of a homogeneous soft layer supported on a rigid substrate. **b** Schematic of wrinkle formation in a typical bilayer system composed of a rigid film on an elastic substrate. *h*_f_ is the film thickness, *A* and *λ* represent the amplitude and wavelength of the wrinkle, respectively. **c**–**f** Schematic of several typical morphological instability: **c** period double, **d** period quadruple, **e** fold, and **f** ridge

The surface instability can be induced in homogenous soft materials [66, 67], as well as bilayer film–substrate systems [68–70]. For a homogenous soft layer confined by a rigid substrate, the compressive stress can be induced by the constrained swelling or volumetric growth, and the induced stress can trigger the surface instability of the soft layer, leading to the formation of creases with sharp self-contact tips in many situations [71–76] (Fig. 2a). The formation of creasing patterns on homogenous soft layers is difficult to be controlled due to its high sensitivity to surface defects [77], and most previous studies focused on the surface instability of film–substrate bilayer systems. For a bilayer consisting of a rigid film bonded on a soft substrate, the compressive stress in the film can be induced by various external stimuli such as prestrain relaxation [78–80], thermal shrinkage [48, 81], and swelling [82–84]. Sufficiently large compressive stress in the film is able to trigger the formation of wrinkles, forming a new equilibrium state.

Generally, two strategies, force balance and energy balance, are used to analyze the wrinkling of thin films in bilayer systems. Consider an elastic thin film within the *FvK* theoretical framework strongly bonded on a semi-infinite substrate (plane-strain condition). When the film is subjected to a uniaxial compressive load, the formation of wrinkles can be induced when the compressive stress in the film reaches the threshold (Fig. 2b). Assuming that the wrinkles have a sinusoidal profile, the compressive stress (*F*) in the film can be calculated by the modulus (*E*) and Poisson’s ratio (*ν*) of the film and the substrate, the thickness (*h*_f_), and the width (*w*) of the film and can be described by Eq. 1 [85, 86]

1 $$F = E_{{\text{f}}} \left[ {\left( {\frac{\pi }{\lambda }} \right)^{2} \frac{{wt^{3} }}{{3\left( {1 - \nu_{{\text{f}}}^{2} } \right)}} + \frac{{\lambda E_{{\text{s}}} w}}{{4\pi \left( {1 - \nu_{{\text{s}}}^{2} } \right)E_{{\text{f}}} }}} \right],$$

where the subscripts *f* and *s* represent the film and the substrate, respectively, and *λ* represents the wrinkling wavelength. The critical wavelength (*λ*_c_) of wrinkles can be derived from d*F*/d*λ* = 0 by minimization of *F* and can be described by Eq. 2:

2 $$\lambda_{{\text{c}}} = 2\pi h_{{\text{f}}} \left( {\frac{{\overline{{E_{{\text{f}}} }} }}{{3E_{{\text{s}}} }}} \right)^{1/3} ,$$

where $\overline{E} = E/(1 - v^{2} )$ is the plane-strain modulus. Equation 2 indicates that the critical wavelength of wrinkles is determined by the modulus ratio of the film to the substrate and the film thickness, and stiffer film on softer substrate favors larger wrinkling wavelength. Based on the assumption that *λ* is independent of applied strain *ε*, the amplitude *A* of wrinkles can be predicted by the film thickness *h*_f_, applied strain *ε*, and the critical strain *ε*_c_ for the onset of wrinkles and can be described by Eq. 3:

3 $$A = h_{{\text{f}}} \sqrt {\frac{\varepsilon }{{\varepsilon_{{\text{c}}} }} - 1} ,$$

where $\varepsilon_{{\text{c}}} = (1/4)(3\overline{{E_{{\text{s}}} }} /\overline{{E_{{\text{f}}} }} )^{2/3}$ only depends on the modulus ratio of the film to the substrate. From the standpoint of energy, the wrinkling of thin films on soft substrates is also a process to seek a balance between the bending energy of the stiff film and the deformation energy of the substrate [87–89]. For a thin stiff film bonded on a thick compliant substrate, surface wrinkling can be induced to release the total system energy under sufficient in-plane compressive stress, and same predictions as described by Eqs. 2 and 3 can be obtained by the energy-balance method [89].

However, the above-mentioned mechanics models based on linear stability analysis under small deformation is only effective to the initial stage of wrinkling. The wrinkling wavelength in Eq. 2 is independent of the prestrain of the substrate while the experiments showed that the wavelength decreases with increasing the compression of the film [90, 91]. The determination of the wavelength and the amplitude of wrinkles under large deformation requires non-linear analysis. Considering a wrinkled film subjected to an external applied strain (*ε*_applied_), Jiang et al. [90] developed a mechanics model for the prediction of the wavelength and the amplitude of wrinkles under finite deformation which can be described by Eqs. 4 and 5:

4 $$\lambda = \frac{{\lambda_{{\text{c}}} (1 + \varepsilon_{{{\text{applied}}}} )}}{{(1 + \varepsilon_{{{\text{pre}}}} )(1 + \varepsilon_{{{\text{applied}}}} + \zeta )}},$$

5 $$A = h_{{\text{f}}} \frac{{\sqrt {(\varepsilon_{{{\text{pre}}}} - \varepsilon_{{{\text{applied}}}} )/\varepsilon_{{\text{c}}} - 1} }}{{\sqrt {1 + \varepsilon_{{{\text{pre}}}} } (1 + \varepsilon_{{{\text{applied}}}} + \zeta )^{1/3} }},$$

where *λ*_c_is the critical wrinkling wavelength, *ε*_pre_ is the prestrain of the substrate, and $\zeta = 5\varepsilon_{{{\text{pre}}}} (1 + \varepsilon_{{{\text{pre}}}} )/32$. Equations 4 and 5 provide quantitative predictions for the wavelength and the amplitude of wrinkles under external applied strain. However, this finite deformation model is only effective to the instability mode of the wrinkle. The determination of other post-wrinkle morphologies requires non-linear analysis based on much larger deformation.

Most previous studies on postbuckling analyses concentrated on numerical simulations and experimental fabrications while a few exact solutions were achieved for simple cases [92–96]. For example, Pocivavsek et al. [97] observed that the preformed sinusoidal wrinkles on a fluid could transform into a large fold by further increasing the compression of the film. By replacing the liquid to an elastic substrate, Brau et al. [98] demonstrated that the evolution of the wrinkle into distinctly different morphologies can be induced by progressively compressing the film. They found that the sinusoidal wrinkles first transformed into period doubles (Fig. 2c) that further evolved into period quadruples (Fig. 2d) or folds with self-contact valleys (Fig. 2e) with increasing the compressive strain. By accounting for a stress-free film bonded on a prestretched elastic substrate, Cao and Hutchinson [99] predicted that sufficiently large prestrain could trigger the formation of ridges with sharp peaks (Fig. 2f). Besides, Kim et al. [100] demonstrated that the transition from two-dimensional wrinkles to hierarchical folds can be induced by continuous biaxial compression, where the film was divided into a number of domains by the dominated folds, leading to the formation of networks. Rich morphology evolution of wrinkles has fascinated scientists to investigate the transition mechanisms among various surface instability modes.

Depending on the film–substrate modulus ratio and film thickness, the initial instability modes can be either wrinkles or creases [101, 102]. Generally, stiffer and thinner film tends to form wrinkles under small compression while softer and thicker film tends to form creases under large compression [102]. By considering a stress-free film bonded on a prestretched elastic substrate, Wang and Zhao [31, 103] reported a 3D phase diagram for the description of wrinkling morphology evolution with the variation of shear modulus ratio, normalized adhesion energy, and the mismatch strain. However, the prestretch of the substrate has been demonstrated to have significant effect on postbuckling morphology [104–106], postbuckling analysis based on un-stretched substrates is critical for understanding buckling behaviors of the bilayer with a modest contrast in modulus [103, 107–109]. For example, understanding the morphology evolution of biological organs composed of multiple soft layers requires bilayer models with small film–substrate modulus ratio. Tallinen et al. [107] simulated the morphology evolution of brain cortex based on a soft bilayer with similar modulus under differential growth. Kim et al. [108] investigated the morphology evolution of wrinkles in bilayer systems with small modulus ratio and demonstrated three types of post-wrinkling bifurcations. Auguste et al. [109] systematically studied the evolution of wrinkles into creases in elastic bilayers with modest contrast in modulus, and they observed two typical post-wrinkle bifurcations: wrinkles directly transform into creases, and wrinkles transform into period doubles first and then evolve into creases. Besides, Hayward et al. [110] demonstrated that the formation of high-aspect-ratio wrinkles and ridges can be induced in film–substrate bilayer with small thickness contrast.

Beyond the wrinkling of large-size uniform film bonded on elastic substrate, recently, a series of studies have revealed differently post-wrinkle morphologies in systems composed of patterned stiff films on soft substrates [111–119]. When the length scale of the pattern is comparable to the wavelength of wrinkles, stress localization near the pattern arises under compression. The feature size, geometry, and spacing are all demonstrated to have important effect on the wrinkling and post-wrinkle morphologies of patterned films [111, 114, 116, 118]. For example, the preformed pattern can act as novel boundary to direct surface wrinkling for controllable fabrication of hierarchical wrinkling patterns with precise orientation [113, 119]. Besides, stress localization near the patterned region triggers the decrease of the threshold compression for the onset of various wrinkling morphologies. For example, Wang et al. demonstrated that the critical strains for initializing the wrinkle-to-crease transition at the edge of lattice hole are much lower than the typical critical strain value [116]. By considering both the imposed stiff patterns and the soft bare substrate, Ouchi et al. [118] studied the morphology evolution of a non-continuous patterned stiff film bonded on a soft substrate under uniaxial compressive stress. Depending on the ratio of stripe length to wrinkle wavelength and gap size, they observed a set of surface morphologies including wrinkling, period-doubling, Euler buckling, zigzag, and sawtooth patterns.

With introducing biaxial stress in the film–substrate system, a thin film can buckle into various wrinkling patterns such as triangular, checkerboard, hexagonal, herringbone, and labyrinths patterns [92–95, 120–125]. The selection and transition of these morphologies strongly depend on the stress state and the loading sequence of the stress. Huang et al. [122] demonstrated that the critical strain for checkerboard pattern is slightly higher than that of wrinkles, and then the checkerboard can transform into herringbone pattern with increasing the compression. Under moderate equi-biaxial stress, thin stiff films on elastic substrates tend to buckle into herringbone pattern to minimize the system energy [92, 124]. When the loading stress is equi-biaxial, the critical compressive stress *σ*_c_ and the critical strain *ε*_c_ for the onset of wrinkles can be given by Eqs. 6 and 7 [93, 124]:

6 $$\sigma_{{\text{c}}} = \frac{{\overline{{E_{{\text{f}}} }} }}{4}\left( {\frac{{3\overline{{E_{{\text{s}}} }} }}{{E_{{\text{f}}} }}} \right)^{\frac{2}{3}} ,$$

7 $$\varepsilon_{{\text{c}}} = \frac{1}{{4(1 + \nu_{{\text{f}}} )}}\left( {\frac{{3\overline{{E_{{\text{s}}} }} }}{{\overline{{E_{{\text{f}}} }} }}} \right)^{\frac{2}{3}} .$$

Zhao et al*.* [126] investigated the effect of lateral dimension on the wrinkling of a thin film on compliant substrate under differential growth/swelling. A square film is considered as an elastic plate subjected to isotropic growth or swelling-induced biaxial compression, which is bonded on a semi-infinite elastic substrate. The width of the film is 2*a* and the thickness is *h*_f_. The critical growth strain *ε*_g_ for initiating the wrinkling of the square film can be given by Eq. 8:

8 $$\varepsilon_{{\text{g}}} = \frac{1}{{4(1 + \nu_{{\text{f}}} )}}\left( {\frac{{3\overline{{E_{{\text{s}}} }} }}{{E_{{\text{f}}} }}} \right)^{\frac{2}{3}} + \frac{3}{8}\frac{{\lambda_{{\text{c}}} }}{2a},$$

where *λ*_c_ is the critical wrinkling wavelength. Besides the modulus ratio, Eq. 8 suggests that the critical growth strain for the wrinkling of the square film also depends on the ratio of the critical wavelength to the film width. When the width of the film is comparable to the wavelength, the onset of wrinkling may need much higher critical strain than that for the film with infinite width.

Beyond the well-bonded film–substrate system, the non-coplanar mesh design composed of islands and interconnects is significant for fabricating 3D buckled structures for stretchable electronics [40, 127, 128]. Generally, interconnects with smaller width are not bonded on the substrate while the islands with larger width are strongly attached on the substrate. By taking the system as beams with clamped ends, Song et al. [129] developed a mechanics model to analyze the buckling of interconnects based on energy method, where the amplitude of buckled interconnects can be given by minimizing the total energy. More detailed information about the mechanics behind the buckled structures based on non-coplanar mesh design is available in recent reviews [32, 33].

### Curved Substrates

#### Core–Shell Cylinders and Tubes

There are two typical instability modes for long cylinders: surface wrinkling without changing the central axis of the cylinder and global buckling with a wavy shape. Here we will focus on surface wrinkling of core–shell cylinders subjected to various load, such as axial stress, circumferential stress, swelling/shrinkage, and volumetric growth.

**Fig. 3:**
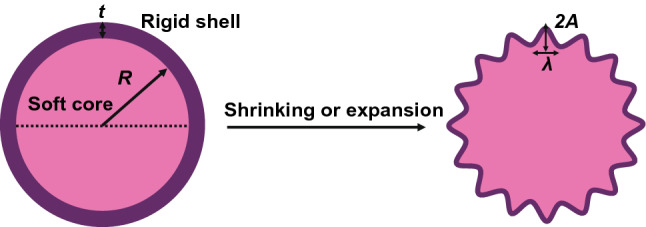
Schematic of wrinkle formation in a typical core–shell system composed of a rigid shell bonded on a soft core under shrinking or expansion. *R* is the radius of the soft core, *t* represents the shell thickness, *A* and *λ* represent the amplitude and wavelength of the wrinkle

For a long cylinder with a radius *R* coated with a thin film with a thickness *t* (*R*/*t* >  > 1), assume the core–shell cylinder a plane-strain system. Circumferential stress (*σ*) in the film can be induced with increasing the mismatch strain (Δ*ε*) between the film and the substrate. When the compressive stress reaches the threshold, the formation of wrinkles can be induced to release the total system energy [52, 130] (Fig. 3). The compressive stress (*σ*_0_) in the film at pre-buckling point can be described as shown in Eq. 9 [49]:

9 $$\sigma_{{0}} = \frac{{E_{{{\text{shell}}}} E_{{{\text{core}}}} (2R^{2} + 2RT + t^{2} )\Delta \varepsilon }}{{2E_{{{\text{core}}}} (1 - \nu_{{{\text{shell}}}}^{2} )R^{2} + [E_{{{\text{core}}}} (1 + \nu_{{{\text{shell}}}} ) + E_{{{\text{shell}}}} 4(1 + \nu_{{{\text{core}}}} )(1 - 2\nu_{{{\text{core}}}} )](2RT + t^{2} )}}.$$

Equation 9 suggests that *σ*_0_ is affected by the substrate curvature, mismatch strain, and the modulus ratio. Mismatch strain between the shell and the core can be induced by inhomogeneous expansion or shrinking induced by external stimuli such as thermal shrinkage [58, 59], solvent-induced swelling [52, 131, 132], and volumetric growth [2, 10, 14]. The mismatch strain can be calculated by the expansion coefficient of the shell and the core, denoted as *α*_shell_ and *α*_core_, respectively. For example, upon heating or cooling, Δ*ε* can be calculated by Δε = |α_shell_ − α_core_| Δ*T*, Δ*T* is the temperature change. Similarly, this equation can be used to calculate mismatch strain induced by swelling or growth. The critical wavelength *λ*_c_, critical stress *σ*_c_, and corresponding wave number (*n*_c_) can be obtained from a simplified plane-strain ring-foundation model and can be described by Eqs. 10–12 [21, 49]:

10 $$n_{{\text{c}}} = \left( \frac{R}{t} \right)^{\frac{3}{4}} \left[ {\frac{{12E_{{{\text{core}}}} (1 - \nu_{{{\text{shell}}}}^{2} )}}{{E_{{{\text{shell}}}} (1 + \nu_{{{\text{core}}}} )(1 - 2\nu_{{{\text{core}}}} )}}} \right]^{\frac{1}{4}} ,$$

11 $$\lambda_{{\text{c}}} = \frac{2\pi R}{{n_{{\text{c}}} }}2\pi t\left( \frac{R}{t} \right)^{\frac{1}{4}} \left[ {\frac{{E_{{{\text{shell}}}} (1 + \nu_{{{\text{core}}}} )(1 - 2\nu_{{{\text{core}}}} )}}{{12E_{{{\text{core}}}} (1 - \nu_{{{\text{shell}}}}^{2} )}}} \right]^{\frac{1}{4}} ,$$

12 $$\sigma_{{\text{c}}} = \left( \frac{t}{R} \right)^{\frac{1}{2}} \left[ {\frac{{E_{{{\text{shell}}}} E_{{{\text{core}}}} }}{{3(1 - \nu_{{{\text{shell}}}}^{2} )(1 + \nu_{{{\text{core}}}} )(1 - 2\nu_{{{\text{core}}}} )}}} \right]^{\frac{1}{2}} .$$

The system curvature in Eqs. 10–12 plays an important role in determining the onset of initial wrinkles. Similar to planar substrates, the wrinkling amplitude *A* of core–shell cylinders can also be predicted through the compressive strain, critical strain, and the film thickness (Eq. 13). Differently, the critical strain (*ε*_c_) for initiating the wrinkling of a thin film on a cylindrical substrate is affected by the substrate curvature obviously. Equation 14 shows that the critical strain increases with the curvature (*t*/*R*), indicating that the curvature delays surface wrinkling.

13 $$A = t\left[ {\frac{2}{3}\left( {\frac{\varepsilon }{{\varepsilon_{{\text{c}}} }} - 1} \right)} \right]^{\frac{1}{2}} ,$$

14 $$\varepsilon_{{\text{c}}} = \left[ {\frac{{E_{{{\text{core}}}} (1 - \nu_{{{\text{shell}}}}^{2} )t}}{{3E_{{{\text{shell}}}} (1 + \nu_{{{\text{core}}}} )(1 - 2\nu_{{{\text{core}}}} )R}}} \right]^{\frac{1}{2}} .$$

Yin et al. [52] demonstrated that a smooth core–shell cylinder can transform into wrinkled gears under circumferential compressive stress. Cao et al. [133] investigated the postbuckling morphology of a core–shell cylinder under the swelling of the stiff shell or the shrinkage of the soft core. They found that a wrinkle-to-fold transition may occur when the deformation (swelling or shrinkage) is far beyond the critical value for wrinkles, leading to the formation of period-doubling morphology. When a core–shell cylinder is subjected to an axial compression, the thin shell may buckle into axisymmetric wrinkling morphology first and then transform into hexagonal mode due to the curvature effect [134, 135]. For a soft cylindrical shell that are slidable on a stiff cylinder, Yang et al. [136] demonstrated that the slidable soft shell subjected to axial compression buckles into wrinkles first and then several wrinkles transform into localized ridge that can further envolve into sagging ridge with increasing the compression. When a core–shell cylinder is subjected to both radial and longitudinal growth, various wrinkling morphologies and post-wrinkle morphologies (e.g., hexagon, labyrinth pattern) can be induced by varying the geometries and stress states [137, 138]. Recently, Zhang et al. [139] investigated the wrinkling morphology evolution on tori with non-uniform curvature by finite element simulations. They found that stripe patterns tend to form on softer cores while hexagonal patterns tend to form on stiffer cores, and hybrid patterns consisting of both hexagons and stripes form on the cores with moderate stiffness. Due to non-uniform curvature, stripe patterns form on the inner surface of the torus, whereas hexagon patterns form on the outer surface at initial wrinkling stage, both of them transform into zigzag and segmented labyrinth patterns with increasing the deformation.

When the length of the cylinder is comparable to or even shorter than the diameter of the cylinder, such core–shell systems satisfy the plane-strain or plane-stress conditions. Zhao et al. [140] obtained a phase diagram for the prediction of wrinkling morphology in curved film–substrate systems. A soft shell bonded on a rigid core subjected to volumetric growth tends to form creasing morphology while a thin stiff shell on a soft core tends to form wrinkles first that can further transform into period doubles, folds, and ridges depending on the core–shell materials properties and the substrate curvature [140–142]. By simplifying the cylindrical core–shell system to a 2D problem of a ring on an annular substrate, Lagrange et al. [143] provided a solution for the hoop stress in the ring with accounting for both the curvature and the finite size of the substrate. Depending on the dimensionless thickness and stiffness ratio, two types of instability modes including local wrinkling of the ring and global buckling of the structure were identified. In such simplified 2D core–shell system, their analysis showed that the critical stress for the wrinkling does not depend significantly on the substrate curvature.

In many situations, tubular tissues composed of multilayers can evolve into various wrinkling morphologies and a number of studies demonstrated that the wrinkling of tubular organs is highly related to smooth muscle contraction, external mechanical loads, and constrained tissue growth [17, 144–150]. Li et al. [17, 148] simulated the morphology evolution of a tubular mucosal layer under confined growth. The simulation results suggested that the wrinkling morphology may evolve towards a period-doubling morphology when continuous growth of mucosa is far beyond the threshold. When the volumetric growth of a tubular tissue is inhomogeneous, the formation of localized wrinkling patterns may occur due to growth-induced non-uniform stress [17, 151]. It is worth mentioning that the formation of axial wrinkling patterns may be energetically beneficial for cylindrical organs growing in the longitudinal direction [152–154].

#### Core–Shell Spheres

Sphere is another typical curved substrate with constant curvature. Consider a soft sphere with a radius *R* covered with an isotropic stiff shell with a thickness *t*. Equi-biaxial stress in the shell can be induced with increasing the mismatch strain between the core and the shell, the compressive stress in the shell at pre-buckling point can be predicted by Eq. 15 [49]:

15 $$\sigma_{{0}} = \frac{{E_{{{\text{shell}}}} E_{{{\text{core}}}} (3R^{3} + 3R^{2} t + 3Rt^{2} + t^{3} )\Delta \varepsilon }}{{3E_{{{\text{core}}}} R^{3} (1 - \nu_{{{\text{shell}}}} ) + E_{{{\text{core}}}} (1 + \nu_{{{\text{shell}}}} )(3R^{2} t + 3Rt^{2} + t^{3} ) + 2E_{{{\text{shell}}}} (1 - 2\nu_{{{\text{core}}}} )t(3R^{2} + 3Rt + t^{2} )}}.$$

Similar to core–shell cylinders, Eq. 15 suggests that the onset of wrinkling patterns on core–shell spheres is also determined by the modulus ratio and the curvature. With increasing the excess stress in the shell layer or varying the substrate curvature, rich postbuckling morphologies can be induced on the surface of core–shell spheres [155–158]. Most postbuckling analyses of core–shell spheres are based on numerical and experimental approaches due to its notorious difficulty [132, 155–159].

Many fruits and vegetables with core–shell structures present novel wrinkling morphologies on their surfaces such as Korean melon, ridged gourd, small pumpkin, dehydrated pollen grains, and dehydrated green peas [21, 155, 159–161]. Yin et al. [21, 159] analyzed the surface instability of various spheroidal core–shell structures based on the theory of thin elastic shell and reproduced the wrinkling morphologies of many fruits and vegetables by finite element simulation. They found that the onset of wrinkling patterns on spheroidal core–shell structures mainly depends on three dimensionless parameters, the ratio of effective size/thickness, the ratio of equatorial/polar radii, and the ratio of core/shell modulus. Li et al. [155] investigated the wrinkling and post-wrinkling morphology of a neo-Hookean core–shell sphere subjected to the shrinkage of the core through theoretical analysis and non-linear simulations. The core–shell sphere shrinks isotropically first and then suddenly buckles into periodic dimples to release the compression in the shell when the shrinkage reaches a threshold. With further increasing the shrinkage, dimple pattern evolves into a pattern consisting of regular pentagons and hexagons. After that, sufficiently large shrinkage triggers a wrinkle-to-fold transition and the dimple pattern transforms into labyrinthine pattern to release more elastic strain energy. The morphology evolution of the core–shell sphere was confirmed by the dehydration of a green pea. Their further analysis showed that the flat-wrinkle-fold morphology transition induced by core shrinking is also a process of seeking energy minimization.

Though a number of simulations of wrinkling morphology evolution on the surface with constant curvature have been realized, simple and general theoretical models and more efficient numerical methods are needed to describe and predict complex wrinkling patterns on the surface with non-uniform curvature.

#### General Theory for the Wrinkling and Pattern Selection in Curved Elastic Bilayer

By reducing the Koiters elastic shell theory to the Swift–Hohenberg equation, Stoop et al*.* [157] developed a general Swift–Hohenberg theory for describing the wrinkling morphology and pattern selection in curved bilayer systems. Their analysis and experiments showed that the substrate curvature plays a significant role in pattern selection, which is consistent with the results of previous studies [132, 155, 156, 158]. Based on the generalized Swift–Hohenberg theory, López Jiménez et al. [162] studied the effect of curvature and topology on crystalline dimpled patterns on curved elastic bilayers. They demonstrated that the defects localization strongly depends on the local Gaussian curvature and its gradients across a surface, which further confirmed that the curvature has important implications on the morphology evolution in curved bilayer systems, since defects localization is critical for secondary bifurcation [163]. Recently, Veldin et al. [164] reported a novel computational model for quantitative prediction of wrinkling patterns on general curved core–shell systems subjected to pressure. Zhao et al. [165] studied the effect of curvature anisotropy and curvature gradient on the wrinkling of curved elastic bilayers and provided a phase diagram describing the pattern selection in curved bilayer systems with anisotropic curvature. They found that the initial instability mode of curved elastic bilayer can also be sinusoidal when the curvature tensor is anisotropic and the wrinkle is perpendicular to the principal curvature. With progressively increasing the excess stress, the sinusoidal wrinkles may evolve into hexagonal pattern that can further transform into labyrinth pattern by experiencing a bistable mode.

#### Importance of the Substrate Curvature

Compared with planar substrate with zero curvature, the wrinkling of thin films on curved substrates has some special features: (i) The critical strain for the wrinkling of thin films on curved substrates increases with the curvature and wrinkling patterns tend to form on substrates with smaller curvatures when other parameters are constant [49, 140, 156, 166]. Alain et al. [166] theoretically demonstrated that curvature delays growth-induced wrinkling compared with flat substrate. Crosby et al. [156] found that wrinkled patterns can be induced on oxide PDMS spheres having big radii under swelling-induced compressive stress, while it is hard to form wrinkles on the spheres with small radii. (ii) The wrinkling morphology highly depends on the substrate curvature [140, 157, 158]. Taking spherical core–shell system as an example, with increasing the effective radius *R*/*t*, hexagonal pattern forms first and then transforms into labyrinth pattern via a bistable mode [157]. Beyond the film thickness, modulus ratio, and mismatch strain, substrate curvature has been demonstrated to be one more significant parameter that is capable of controlling surface wrinkling [157, 158, 167–169]. Curved core–shell structures have more deformation modes than planar bilayers, which make it possible to fabricate novel wrinkling patterns on curved substrates with various geometries. Besides, the deformations usually occur in axial direction for planar bilayers while it can be in normal, tangential, and axial directions for curved core–shell systems. (iii) Different from 2D films on planar substrates, curved substrates can be both solid and hollow with various 3D geometries across multiple length scales. As summarized in Table 1, wrinkling patterns in nature are observed not only on the surfaces of solid spheres, cylinders, and cones, but also on both inner and outer surfaces of hollow spheres and cylindrical tubes. A number of theoretical analysis and numerical simulations have demonstrated the significance of substrate curvature in shaping the biological morphologies such as the development of brain cortex [22, 170], the folding of the mucosa [17, 171–173], and the buckling of plant tissues [21, 155].

**Table 1:** Examples of wrinkling patterns on curved substrates observed in nature and corresponding simulation studies

Context	Geometry	Pattern	Wavelength (m)	Shell/core	Compression	Simulation	Refs
Brain	Sphere	Fold	10^−2^	Cortex/matter	Growth	Yes	[22, 170]
Green pea	Sphere	Wrinkle, ridge	10^−3^	Testa/seed	Dehydration	Yes	[155]
Cactus	Cylinder	Ridge	10^−2^	Epidermis/flesh	Growth	Yes	[140]
Wax apple	Cone	Wrinkle	10^−3^	Epidermis/flesh	Growth	Yes	[21]
Fingertips	Sphere and cylinder	Wrinkle	10^−3^	Epidermis/dermis	Swelling	Yes	[174]
Stomach	Hollow ellipsoid	Crease	10^−3^	Mucosal/muscular	Growth	Yes	[171]
Muscular artery	Cylindrical tube	Double	10^−3^	Mucosal/muscular	Growth	Yes	[140]
Ductus deferens	Cylindrical tube	Ridge	10^−4^	Mucosal/muscular	Growth	Yes	[140]
Colon	Cylindrical tube	Crease	10^−2^	Mucosal/muscular	Growth	Yes	[140]
Bovine esophagus	Cylindrical tube	Crease	10^−3^	Mucosal/muscular	Growth	Yes	[17]
Porcine airways	Cylindrical tube	Wrinkle	10^−3^	Mucosal/muscular	Growth	Yes	[17]
Bacterium	Sphere and cylinder	Wrinkle	10^−7^	Cell membrane/cytoplasm	Osmotic pressure	Yes	[175]
Rose petal	Cone	Fold	10^−8^ to 10^−5^, Multiple	Epidermis/flesh	Growth	No	[18]
Small intestine	Cylindrical tube	Fold	10^−6^ to 10^−3^, Multiple	Mucosal/muscular	Growth	No	[172, 173, 176]
Bronchi	Cylindrical tube	Wrinkle	10^−4^ to 10^−2^, Multiple	Mucosal/muscular	Growth	Yes	[15, 16]
Pumpkin	Spheroidal shape	Ridge	10^−2^	Epidermis/flesh	Growth	Yes	[21]
Cantaloupe	Spheroidal shape	Buckle	10^−3^	Epidermis/flesh	Growth	Yes	[21]

Therefore, substrate curvature not only plays an important role in determining the critical point for the onset of surface wrinkling, but also is capable of controlling the wrinkling morphology evolution. Importantly, the formation of wrinkling patterns on curved substrates by harnessing mechanical instabilities opens new avenues for understanding the wrinkling mechanism of biological surfaces and provides simple approaches for fabricating microstructures on substrates with various geometries.

## Bio-inspired Fabrication of Wrinkling Patterns on Curved Substrates

**Fig. 4:**
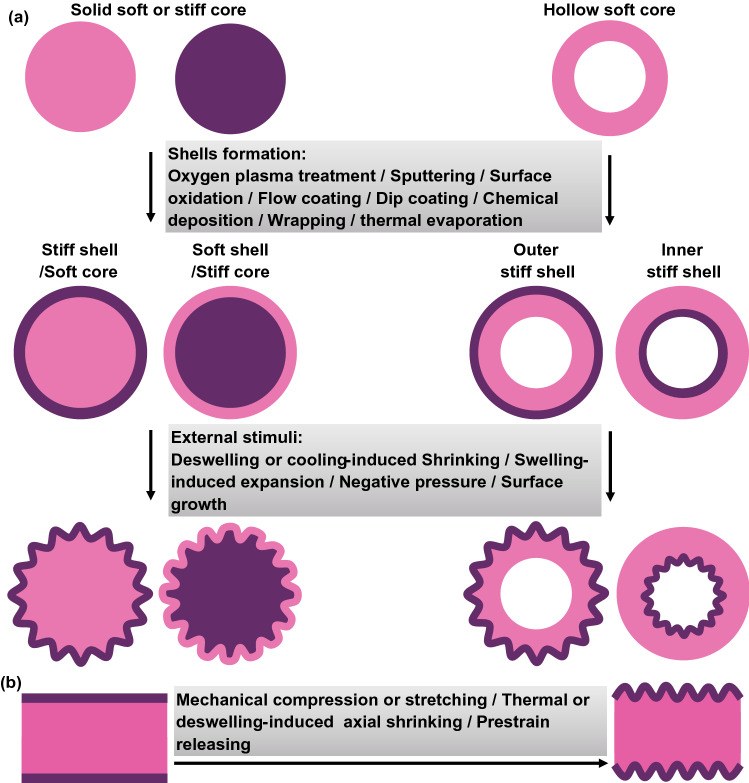
Schematic illustration of various types of core–shell systems for the fabrication of wrinkling patterning on curved substrates. **a** Solid cores with stiff or soft shells and hollow substrates with outer or inner stiff shells. **b** Approaches used to induce axial deformation for ring-patterning on core–shell cylinders

Depending on the geometries of the cores, closed core–shell systems can be divided into two categories: one is based on solid substrates covered with shells, and the other one is composed of hollow substrates and shells (Fig. 4a). For most cases in nature, the formation of core–shell structures and sufficiently large compressive stress in the shells are two essential conditions for the wrinkling of biological surfaces. Similarly, the fabrication of wrinkling patterns on curved substrates can also be realized by constructing core–shell structures with mismatch modulus and then applying external stimuli to trigger surface instability.

As shown in Fig. 4, methods using to obtain core–shell structures include chemical approaches such as surface treatment of oxygen plasma (UV and UVO) [177–180], surface chemical oxidation [132], chemical vapor deposition [58, 181], as well as physical approaches such as metal sputtering [59, 182], dip coating [54, 55, 61], and mechanical wrapping [53, 60, 64]. The formation of wrinkling patterns on curved substrates can be induced by applying various external stimuli such as heating/cooling [58, 59], stretching/releasing [53, 60, 183], inflation/deflation [54, 55, 61], and swelling/deswelling [177, 179]. For core–shell cylinders, axial compression is important for fabricating ring-patterned fibers for diverse applications [53, 60] (Fig. 4b). In this section, typical core–shell systems including core–shell spheres, core–shell cylinders, hollow spheres, and tubes with stiff shells are selected as examples to introduce the fabrication methods for diverse wrinkling patterns on curved substrates.

### Solid Core–Shell Spheres

For curved core–shell systems, compressive stress in the shell can be induced by both the shrinkage of the core and the expansion of the shell. Sufficiently large compressive stress may trigger the formation of wrinkling patterns to release the potential energy of the system such as dehydration-induced wrinkling of the green peas [155] and growth-induced folding of the brain cortex [22].

**Fig. 5:**
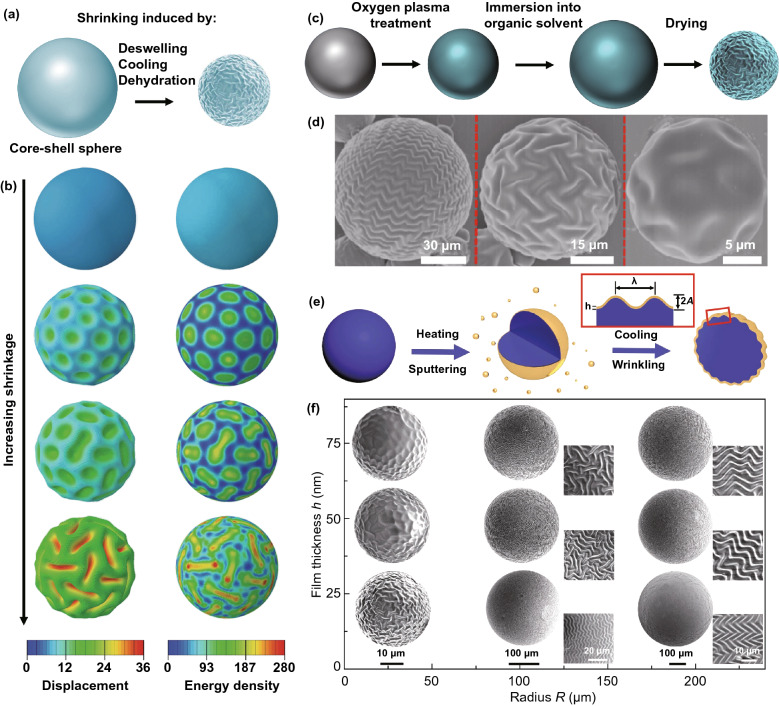
Simulations and experiments of shrinkage-induced surface wrinkling of core–shell spheres. **a** Schematic of the shrinkage of a core–shell sphere under various stimuli. **b** Numerical simulation of the morphology evolution of a core–shell sphere by increasing the shrinkage [155]. Copyright 2011 American Physical Society. **c**, **d** Illustration of the procedure for the wrinkling of PDMS microspheres and SEM images of wrinkled PDMS spheres with different radii [131]. Copyright 2015 American Chemical Society. **e**, **f** Schematic of the fabrication process for metal wrinkles on polymer microspheres and morphology evolution of wrinkling patterns with the film thickness and the microsphere radius [59]. Copyright 2019 Wiley

Shrinkage-induced surface instability of spherical core–shell systems can be realized by thermal, solvents, and chemical stimuli [51, 59, 158] (Fig. 5a). Li et al. [51] prepared inorganic SiO_2_ shells on Ag microspheres by thermal co-evaporation. Due to large difference in thermal-shrinkage coefficient between the shell and the core, compressive stress in the shell can be induced by cooling the core–shell system, leading to the formation of triangular and Fibonacci number patterns. Cao et al. [158] repeated the experiment and observed triangular and labyrinth patterns by varying the radius of spherical substrates suggesting that thermal-induced surface instability of core–shell structures could be a simple approach for the patterning of curved surfaces. However, surface instability patterns such as folds and ridges were not observed on the surface of SiO_2_/Ag microsphere due to limited mismatch strain. Li et al. [155] systematically studied the wrinkling morphology evolution of a core–shell sphere by increasing the shrinkage of the core using finite element simulation. They found that the smooth shell first buckles into symmetrical dimple pattern under small shrinkage and then a wrinkle-to-fold transition was observed with increasing the shrinkage (Fig. 5b). Further analysis revealed that the morphology transition is beneficial for energy stability of the core–shell spheres, and a number of studies reproduced the wrinkle-to-fold (ridge) morphology transition with increasing the shrinkage of the core or excess stress of the shell [156, 157].

Direct surface treatment of soft cores such as oxygen plasma (OP) and chemical oxidation is also a facile approach for the formation of stiff shells on soft cores [131, 132]. Lu et al. [131] fabricated SiO_*x*_ skins on soft PDMS microspheres by OP treatment, and the wrinkling of the stiff shells can be induced by a swelling/deswelling process (Fig. 5c). Multiple parameters including the radius of microspheres, surface treatment time, and the modulus ratio were demonstrated to be able to regulate the wrinkling morphologies (Fig. 5d). Solvent-induced swelling and deswelling of core–shell spheres provide a simple and efficient method for achieving various wrinkling patterns on spherical substrates such as dimples, herringbones, and labyrinth patterns. Beyond thermal deposition and surface treatment, sputtering is also a useful approach to deposit metal shells on soft cores [59, 182]. Recently, Sun et al. [59] obtained tunable wrinkling patterns on metal-coated PDMS microspheres by cooling thermal-expanded PDMS microspheres sputtered with Cr shells (Fig. 5e, f). In addition, they found that controllable friction could be realized by varying the morphology, orientation, and feature size of the wrinkling patterns, which may further extend the application fields of patterned spherical surfaces to flexible electronics, cell culture interfaces, and other biomedical areas [184, 185]. It should be pointed out that the fabrication of surface wrinkles with high aspect ratio on solid core–shell spheres is still a challenge due to limited shrinkage of solid substrates.

**Fig. 6:**
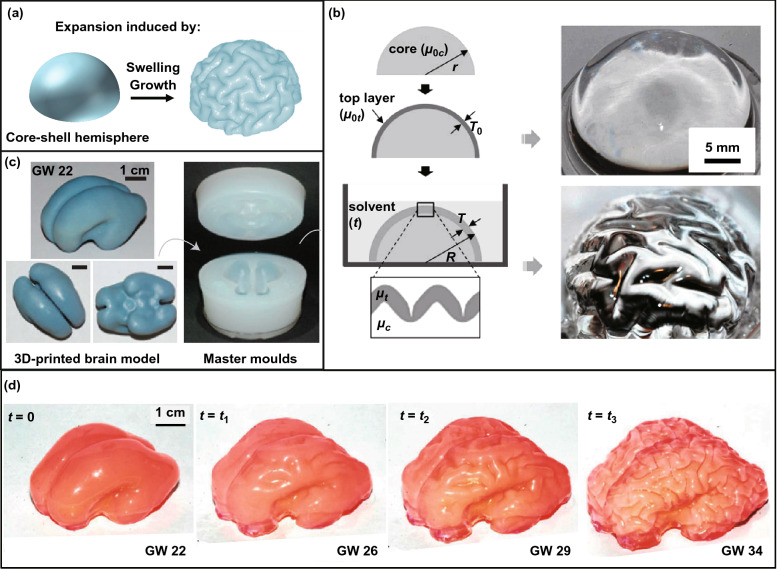
Cortical structures on soft core–shell spheres. **a** Schematic of the folding process of a soft core–shell hemisphere subjected to swelling or volumetric growth [170]. Copyright 2015 American Physical Society. **b** Mimic the morphology of brain cortex through swelling a soft core–shell hemisphere [22]. Copyright 2014 National Academy of Sciences. **c** Images of the 3D-printed brain model and the master molds. **d** Morphology evolution of a replicated gel-brain with increasing the swelling time [65]. Copyright 2016 Springer Nature

Compressive stress in the shells that trigger surface wrinkling in core–shell systems can also be induced by swelling, expansion, and volumetric growth (Fig. 6a) such as the development of brain cortex, and the folding of mucosa [7, 8, 10, 14, 17]. From the standpoint of biology, radial and lateral expansion of the brain can be observed with the differentiation, proliferation, and migration of various types of glial cells, and compressive stress in the cortex can be induced when the cortical layer grows faster than the inner core according to the differential growth theory [186, 187]. The morphology development of brain cortex resulted from non-uniform expansion of the core and the shell has been demonstrated by a number of numerical simulations and experimental studies [9, 22, 65, 170, 188]. Tallinen et al. [22] reported a facile method to mimic the morphology evolution of the brain cortex via swelling a soft core–shell hemisphere (Fig. 6b). With depositing a comparatively stiffer PDMS shell on a softer PDMS hemisphere and then putting the obtained PDMS core–shell hemisphere into organic solvents, swelling-induced tangential stress can trigger the creasing of the shell layer, forming highly convoluted pattern consisting of cusped sulci and smooth gyri. To further mimic the morphology evolution of brain cortex, they obtained a smooth brain mold by 3D-printed method by using a 3D MRI image of a brain of a fetal as the template [65], and a smooth gel-brain made from soft PDMS can be achieved using the obtained mold (Fig. 6c). Similarly, highly convoluted pattern that is extremely similar to the morphology of real brain cortex can be fabricated by immersing the gel-brain coated with stiff shell into organic solvents (Fig. 6d). However, there are still some challenges in the fabrication of cortical structures with higher gyrification index (GI). Besides, the obtained cortical structure is lack of functionalization, which limits their applications in artificial intelligence and the storage of massive information. Although the formation of cortical structures is regulated by complex parameters including genetic, biochemical, and mechanical factors, accumulating evidence indicates that mechanical mismatch plays a significant role in shaping the morphology evolution of brain cortex [188].

### Solid Core–Shell Cylinders

**Fig. 7:**
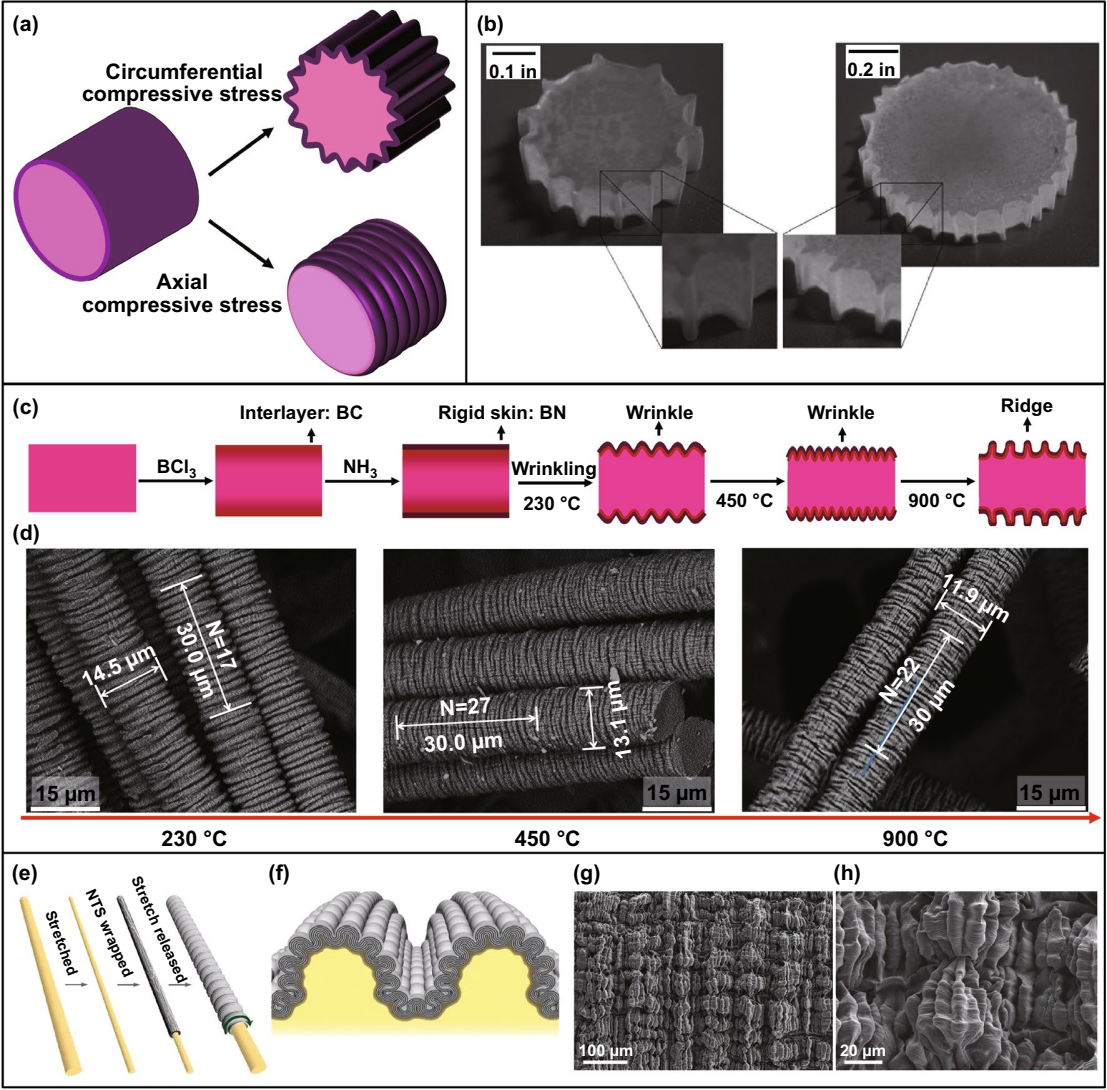
Wrinkling patterns on solid core–shell cylinders under various deformation modes. **a** Schematic of compressive stress in axial and circumferential directions of core–shell cylinders. **b** Images of the self-assembled gears [52]. Copyright 2009 Royal Society of Chemistry. **c**, **d** Schematic of the fabrication process starting from the PAN precursor fibers and SEM images of the wrinkled BN shells [58]. Copyright 2019 Elsevier. **e**–**h** Fabrication process and surface morphology of hierarchically buckled shell–core fibers: **e** schematic diagram of the fabrication process, **f** cross-sectional illustration of hierarchically buckled shell–core fibers, **g**, **h** SEM images of hierarchical buckles at 100% applied strain [53]. Copyright 2015 The American Association for the Advancement of Science

Different from isotropic core–shell spheres, the compressive stress in anisotropic cylindrical shells can be circumferential and axial, and various surface instability modes can be induced under different compressive loads [52, 58, 60] (Fig. 7a). By using polyurethane cylinders as the cores and thin polyvinyl chloride films as the shells, a set of micro-gears can be achieved by dehydration-induced circumferential compressive stress. Besides, the teeth number of the gears can be regulated by varying the core–shell modulus ratio and the substrate curvature [52] (Fig. 7b). Similar axial grooves were observed on stretched polymer microfibers due to the stretch-induced radial shrinkage of the fiber. It is believed that ordered microgrooves on the fibers are beneficial for enhancing the tensile strength [189]. Recently, we fabricated circumferential boron nitride (BN) wrinkles and ridges on microfibers with using polyacrylonitrile (PAN) fibers as precursors [58] (Fig. 7c). Thin BN coating was prepared by chemical vapor deposition of BCl_3_ and NH_3_, and symmetric circumferential wrinkles on the microfibers can be induced via thermal-shrinking-induced axial compressive stress. In addition, a wrinkle-to-ridge transition was observed by increasing the post-treatment temperature from 230 to 900 °C (Fig. 7d).

Prestrain releasing is another efficient approach for triggering circumferential wrinkling patterns on core–shell cylinders [53, 60, 183]. However, an elastic cylinder may contract radially under axial stretching due to Poisson effect. As a result, a thin stiff shell bonded on a prestretched fiber may crack into pieces after releasing the prestrain due to radial expansion of the fiber. To reduce the effect of Poisson ratio, one-dimensional (1D) materials such as carbon nanotubes (CNTs) and Ag nanowires (Ag NWs) combined with elastic coatings are preferentially selected as the shell materials for the fabrication of circumferential wrinkles on elastic fibers [190–192]. Liu et al. [53] fabricated conductive hierarchical buckles on a superelastic fiber by releasing a prestretched rubber fiber wrapped with CNT sheets (Fig. 7e–h). Hierarchical buckles in axial and circumferential directions are observed simultaneously on the fiber under an elongation of 100%. Recently, they constructed periodic self-contact CNT creases on a superelastic rubber fiber by introducing an intermediate soft layer between the outer stiff CNT sheets and the inner elastic fiber, and the contact area between neighboring CNT creases can be regulated by applying external strain [60]. Beyond experimental fabrications, many numerical simulations of wrinkling morphology evolution in cylindrical core–shell systems under various stress states were reported [133, 134, 137, 193]. When a cylindrical shell is subjected to circumferential compressive stress induced by radial shrinkage of the core, axial wrinkles form first and then transform into folds and doubles by increasing the shrinkage [133]. For a core–shell cylinder subjected to axial compression, the symmetry of wrinkling morphology of the cylindrical shell depends on the modulus ratio of the shell to the core [134]. Jia et al. [137] simulated the wrinkling morphology evolution in a cylindrical core–shell system under continuous volume growth. They found that hexagonal pattern could transform into labyrinth patterns by increasing the growth and the morphology is sensitive to the substrate curvature.

**Fig. 8:**
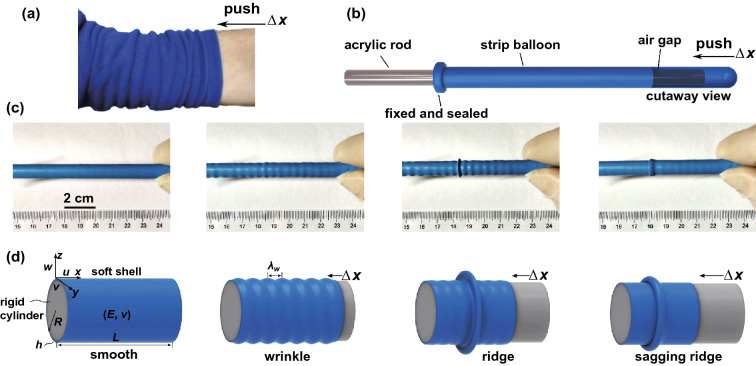
Surface buckling of a soft cylindrical shell supported by a stiff cylinder. **a** Buckling structures observed when a sleeve is pushed up. **b** Experiments of a slidable latex balloon on an acrylic rod. **c**, **d** Photographs and schematic show the morphology evolution by increasing the compression from the right side: initial configuration, wrinkle, ridge, sagging ridge, respectively [136]. Copyright 2018 American Physical Society

For soft layers supported by stiff substrates, surface instability can be induced by constrained volumetric growth [74–76, 194] and axial compression of the soft layers [136]. In such systems, the stiff substrates mainly act as supporting and confining roles in the wrinkling of soft layers. Li et al. [194] simulated the wrinkling morphology of a soft polymer coating covered on a stiff fiber under external electric field through linear stability analysis. They found that ordered wrinkling patterns could be induced through the competition among surface tension, van der Waals and electrostatic interaction. When a flexible tubular shell is slidable on a stiff cylinder, the wrinkling of the tubular shell can be induced by axial compression. For example, a flat sleeve transforms into ridges when we push the soft sleeve along the arm (Fig. 8a). Recently, Yang et al. [136] systematically studied the buckling and postbuckling of cylindrical flexible shells by increasing axial sliding compression. By wrapping a columnar latex balloon on an acrylic rod (one side of the balloon is fixed) and then applying axial compression from the free side, the formation of axisymmetric wrinkles can be induced (Fig. 8b, c). Interestingly, a few wrinkles transform into a localized ridge that can further evolve into a sagging ridge by increasing the compression (Fig. 8c, d). Besides, they analyzed the energy distribution in soft wrinkles, ridges and folds, and explored the critical strains for the formation of various types of wrinkling morphology under certain shell thickness and curvature. The mechanics behind the wrinkling of soft shells on rigid cylinders may provide inspirations for the patterning of other curved soft shells.

### Hollow Spheres and Cylindrical Tubes

For hollow stiff substrates, most previous studies concentrated on the failure analysis of steel tubes under axial compression or bending deformation [195–198], and the wrinkling of soft shells attached on hollow rigid substrates is similar to that of solid systems [136, 194]. Therefore, surface wrinkling of thin stiff shells on hollow soft substrates will be the center of this section. Compared with solid core–shell systems, the expansion and contraction of hollow soft spheres and cylindrical tubes composed of elastic walls can be induced by inflation and deflation, and the contraction-induced compressive stress in the shells can be regulated by varying the pressure difference [54, 55, 61, 157, 199].

**Fig. 9:**
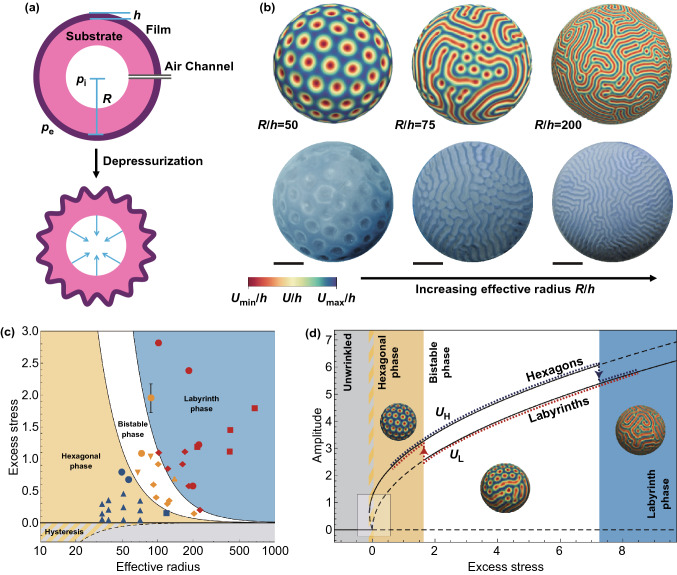
Simulations and fabrications of wrinkling patterns on hollow elastic core–shell spheres. **a** Schematic illustrates the process for the wrinkling of a hollow elastic sphere coated with a thin film. *R* and *h* represent the outer radius of the sphere and the film thickness, *p*_i_ and *p*_e_ represent the inner and outer pressure. The compressive stress can be controlled by tuning the pressure difference, Δ*p* = *p*_e_ − *p*_i_. **b** Simulated wrinkling patterns based on steady-state solutions and fabricated wrinkling patterns demonstrate the hexagonal-to-labyrinth transition via a bistable state with increasing effective radius *R*/*h*. Scale bars 10 mm. **c** Phase diagram of wrinkling morphologies. Experimental data points for hexagonal (blue), bistable (yellow), and labyrinth (red) patterns are shown in the graph, and solid lines are theoretically predicted phase boundaries. **d** Bifurcation diagram of wrinkling patterns, where *R*/*h* = 40, solid and dashed lines represent stable and unstable amplitude solutions respectively [157]. Copyright 2015 Springer Nature. (Color figure online)

For hollow core–shell systems with thick walls (≥ 10 mm), directly pumping the air from the cavity is an efficient way to generate homogeneous compression in the shell, and sufficiently large compressive stress is able to trigger surface instability. For example, by pumping the air from the cavity of a core–shell elastic sphere, the formation of wrinkling patterns can be induced by increasing the internal–external pressure difference (Δ*p*) [157, 199] (Fig. 9a). Norbert et al*.* [157] developed a generalized Swift–Hohenberg theory that is effective to describe the wrinkling morphology and pattern selection in curved elastic bilayers. They demonstrated that a hexagonal-to-labyrinth morphology transition via a bistable phase can be induced by decreasing the curvature at a constant excess stress or increasing the excess stress at a constant curvature (Fig. 9b–d), which is consistent with the results of previous studies [132, 155, 156, 158]. However, negative-pressure-induced compression of thin shells on hollow spheres is small due to limited air volume in the cavity, which may limit their applications for the fabrication of functional films with high area-compression ratio.

**Fig. 10:**
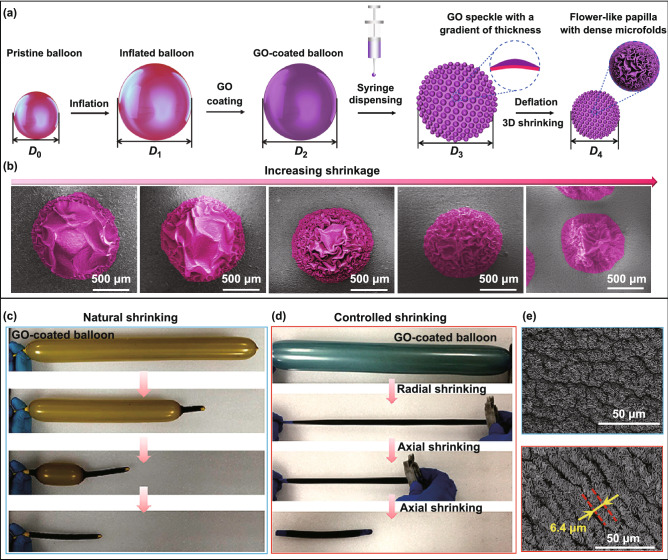
Fabrications of highly convoluted patterns on hollow core–shell spheres and core–shell cylinders by inflation/deflation approach. **a** Schematics of the fabrication process for wrinkled papillae. **b** SEM images with fake colours showing the wrinkling morphology evolution of the papillae with increasing the shrinkage [54]. Copyright 2019 Wiley. **c**, **d** Fabrication of various hierarchical graphene oxide (GO) ridges on tubular balloons by natural shrinking and controlled shrinking. **e** SEM images show random and ordered hierarchical ridging patterns under natural shrinking (blue) and controlled shrinking (red) [61]. Copyright 2019 American Chemical Society. (Color figure online)

For hollow core–shell systems with thin walls (≤ 1 mm), the prestrain of the elastic substrate can be regulated in a wide range by controlling the air amount or liquid volume in the cavity. By coating a thin film on an inflated sphere, deflation-induced global compression can trigger surface instability of the core–shell sphere, leading to the formation of diverse wrinkling patterns. As a cheap commercial product with superelasticity and hollow structure, latex balloons with various geometries have been demonstrated to be suitable curved substrates for the fabrication of highly convoluted films with ultrahigh area-compression ratio [54, 55, 61, 62, 64, 200, 201]. By choosing spherical and cylindrical latex balloons as the substrates, Tan et al. [54, 55, 61] developed a general three-dimensional shrinking method (3DSM) for the self-assembly of 2D graphene oxide (GO) film into 3D complex structures on curved substrates such as cortex-like folds, multiscale ridges, and hierarchically wrinkled papillae. Recently, this method has been extended to the self-assembly of other low-dimensional materials such as CNTs and MXene into complex 3D structures for diverse applications [62, 64]. By dispensing periodic GO speckles on a GO-coated inflated balloon, Tan et al. [54] fabricated hierarchically wrinkled papillae on a spherical substrate through the template-free 3DSM (Fig. 10a). Besides, the microstructures of the papillae can be regulated simply by varying the prestrain of the balloon substrate (Fig. 10b). The formation of 3D-structured papillae from 2D materials by controlled wrinkling on spherical substrate suggests that mechanical self-assembly is a potential approach for the fabrication of 3D architectures on curved substrates. Song et al. [61] demonstrated that the formation of different morphologies can be induced under multiple shrinking modes of tubular balloons. They found that random and oriented ridges can be induced under natural and controlled shrinkage, respectively (Fig. 10c–e). For a GO-coated inflated tubular balloon whose left side is fixed, simultaneous axial and hoop compressive stress in the film can be induced under natural shrinking, forming random-distributed ridges (Fig. 10c, e). By contrast, oriented ridges can be obtained by controlling the balloon radial shrinking first and then axial shrinking (Fig. 10d, e). Besides global compression, compressive stress in curved shells can also be induced by localized point loading, and radial-distributed wrinkling patterns can be achieved due to inhomogeneous deformations [202–204].

**Fig. 11:**
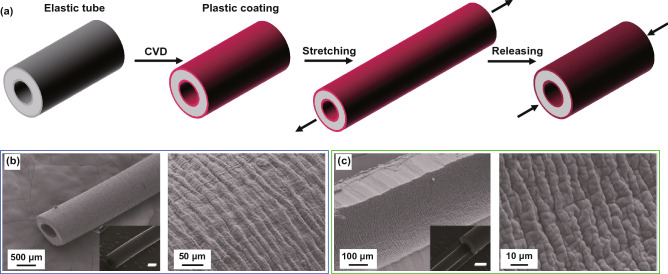
The fabrication of wrinkling patterns on the inner surface of a cylindrical tube.** a** Schematic of the fabrication process for wrinkling patterns on internal surface of a silicone rubber tube: plastic coating on inner and outer surface of the tube can be obtained via chemical vapor deposition, and then wrinkling patterns on the exterior and the interior surface can be induced via stretching and then releasing the plastic/elastic bilayer. The inner and outer diameters of the tube are 0.5 and 1 mm, respectively. Insets of **b** and **c** represent the smooth tube before wrinkling and the scale bars in the insets are 500 μm [181]. Copyright 2016 American Chemical Society

For the wrinkling of internal surfaces in hollow curved systems, most previous studies mainly focused on theoretical analysis and numerical simulations [140, 171–173] while few experiments were reported [181]. To mimic the morphology evolution of mucosa subjected to volumetric growth, Feng et al. [171–173] analyzed and simulated the wrinkling of thin shells bonded on the inner surfaces of hollow spheres and cylindrical tubes. They found that the wrinkling of internal surfaces in hollow curved layered systems is determined by the growth degree, modulus ratio, and substrate curvature. In some situations, the wrinkling of internal surface is sensitive to the internal pressure and surface tension [173]. Zhao et al. [140] studied the wrinkling morphology of stiff shells on concave and convex substrates. They obtained a set of phase diagrams for the prediction of wrinkling morphology evolution in cylindrical core–shell systems, which provides a guidance for the fabrication of wrinkling patterns on the inner surface of hollow curved substrates. Besides, the analysis showed that the substrate curvature is capable of delaying the wrinkling when the shell–core modulus ratio is higher than 100. The fabrication of wrinkling patterns on the inner surfaces of inflated elastic substrates becomes difficult, because it is hard to deposit thin films on the internal surfaces of inflated substrates. By depositing a plastic film (Parylene N) on the internal surface of an elastic silicone tube and then stretching the obtained plastic/elastic bilayer, Takei et al. [181] demonstrated that the wrinkling of the plastic film can be induced after releasing the stretched bilayer since the plastic film could not return to its initial length after releasing the strain (Fig. 11). Besides, they found that this wrinkling technique based on plastic deformation is suitable for the fabrication of high-aspect-ratio ridge structures. However, this method is not valid for the wrinkling of stiff films with poor stretchability because the stiff film may crack into pieces under stretching. Therefore, to develop a simple method for the formation of stiff films on the inner surfaces of inflated substrates is crucial for the patterning of inner surfaces in hollow core–shell systems.

By decreasing the curvature (*h*/*R*) or increasing the excess stress, the wrinkling morphology evolution of stiff shells on hollow substrates with thick walls (≥ 10 mm) is similar to that of solid core–shell systems. Differently, the stiff shells can be bonded on the inner walls or outer surfaces of the hollow substrates while rigid skins only can be attached on the external surface of the solid substrates. Besides, hollow spheres and tubes are suitable substrates for the biofabrication of diverse wrinkling patterns observed on the inner walls of tubular biological organs. Moreover, hollow substrates with thin elastic walls (≤ 1 mm) are capable of providing large deformations by inflation/deflation process, enabling more novel types of wrinkling patterns under giant mismatched strains.

### Other Curved Microarchitectures

Microarchitectures with wrinkled surfaces are ubiquitous in nature such as periodic folded papillae on the rose petal [18], and ridged cones on the leaves of pitcher plant [205]. These beautiful microstructures play significant roles in regulating the surface-wetting properties of biological surfaces. As a lithography-free approach, controlled wrinkling based on surface instability is suitable for the patterning of various microarchitectures such as micropillars, microspheres, microcones, microwells, and other complex microstructures [82, 185, 205, 206].

**Fig. 12:**
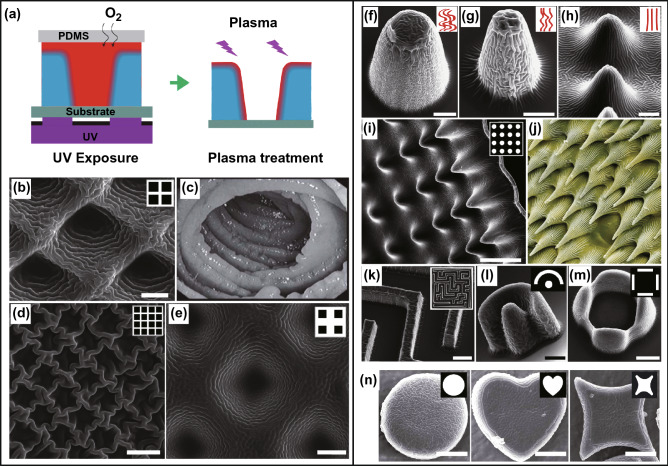
Fabrication of in-plane and out-of-plane wrinkled microarchitectures. **a** Schematics of the fabrication process. **b**–**e** SEM images of wrinkled microwells: **b** microwells whose topography is similar to **c** the jejunum, **d** microwells with wrinkled walls, **e** conical wrinkled microwells. Scale bars are 10 µm. **f**–**n** SEM images of out-of-plane wrinkled microarchitectures: **f**, **g** micropillars with disordered wrinkles, **h**, **i** microcones with ordered wrinkles, **j** surface morphology of the yellow pitcher plant, **k**–**n** wrinkled architectures with various geometries. Scale bars are **f** 30 µm, **g**, **l**–**n** 20 µm, **k** 50 µm, and **h** 10 µm. **a**–**m** Adapted with permission from [205]. Copyright 2015 Wiley. n Adapted with permission from [185]. Copyright 2016 Springer Nature

By combining lithography and controlled wrinkling, Li et al. [185, 205] fabricated a number of in-plane and out-of-plane wrinkled microstructures (Fig. 12). Firstly, microarchitectures with various geometries can be achieved by selective UV exposure of poly (ethylene glycol) diacrylate (PEG-DA) prepolymer, and partially cured-polymer (PCP) layers on the surfaces of the cured microstructures can be obtained by controlling the oxygen diffusion (Fig. 12a). Then surface wrinkling of the PCP-covered microarchitectures can be induced by plasma treatment, forming hierarchical microstructures (Fig. 12b). By adjusting the shapes and sizes of the photo-masks and altering the UV exposure time, various wrinkled in-plane microstructures and out-of-plane microarchitectures can be achieved, such as microwells with different shapes, pillars, cones, labyrinths, and fences (Fig. 12). By deswelling the swollen PDMS micropillars coated with Ag, Gao et al. [82] fabricated wrinkled conductive micropillars for ultrasensitive pressure sensors. These results suggest that controlled wrinkling could be an efficient tool for post-patterning of complex microarchitectures to achieve hierarchical microstructures, which has promising applications in 4D printing, bionic engineering, and artificial organs [207, 208].

Materials selection, core–shell structures formation, and external stimuli using to induce surface instability are all significant for the fabrication of wrinkling structures on curved substrates. Table 2 summarizes main approaches and key parameters for the fabrication of diverse wrinkled structures on various curved substrates and their applications. Soft polymers that are sensitive to mechanical, thermal, and chemical stimuli are selected as the core materials. For example, PDMS spheres [22, 131, 132] and superelastic cylindrical fibers [53, 60, 190–192] are widely used as solid cores while latex balloons are suitable for hollow substrates [54, 55, 61, 62, 64]. Depending on the core materials, the shell materials can be polymers [52, 65], metals [59, 82, 182], and low-dimensional materials including CNTs [53, 60, 192], AgNWs [190, 192], GO [54, 55, 61], BN [58], and MXene [62]. Core–shell structures can be obtained by direct surface treatment [131, 132], chemical vapor deposition [58], sputtering [59, 182], wrapping [53, 60, 64], and dip coating [54, 55, 61]. A number of paired external stimuli such as heating/cooling [58], swelling/deswelling [131, 132], stretching/releasing [53, 60], and inflation/deflation [54, 55, 61] are capable of triggering the wrinkling of films on curved substrates. By controlling the modulus ratio, substrate curvature, and mismatch strain, diverse wrinkling patterns such as dimple, herringbone, labyrinth patterns, as well as wrinkled architectures on curved substrates can be achieved for applications of flexible electronics [53, 60, 190–192], controllable wetting [54, 58], cell culture interfaces [185, 205], and strong actuators [55].

**Table 2:** Main approaches and parameters for the fabrication of diverse wrinkling patterns on various curved substrates and their applications

Systems	Core material	Shell material	Core radius	Shell thickness	Shell formation	External stimuli	Resulted morphology	Potential application	Refs
Stiff shell/soft sphere	PS	Pt	0.2–5 μm	2–13 nm	Sputtering	Swelling	Dimple, labyrinth		[182]
	Ag	SiO_2_	2–8 μm	~ 150 nm	Evaporation	Cooling			[158]
	PDMS	SiO_*x*_	4–15 μm	NA	Chemical oxidation	Deswelling			[132]
	PDMS	SiO_*x*_	5–60 μm	12–13 nm	Oxygen plasma	Deswelling	Dimple, herringbone, labyrinth		[131]
	PDMS	Cr	15–300 μm	25–75 nm	Sputtering	Cooling		Controllable friction	[59]
	PDMS	PDMS	~ 11 mm	0.3–1.2 mm	Surface polymerization	Swelling	Gyrification		[22]
Stiff shell/soft cylinder	PAN	BN	3 ~ 8 μm	~ 31 nm	Chemical vapor deposition	Heating	Ring-like patterns	Tunable wetting	[58]
	Lycra fiber	SEBS/AgNWs/MWCNTs	~ 15 μm	NA	Spray coating	Prestrain releasing	Self-contact creases	Underwater wearable electronics	[190]
	PU fiber	AgNWs	~ 50 µm	6 ± 4 µm	Brush coating	Prestrain releasing	Self-contact creases	Piezoresistive fibers	[191]
	SEBS fiber	CNTs	20–225 µm	~ 10^2^ nm	Mechanical wrapping	Prestrain releasing	Hierarchical buckles	Wearable electronics	[192] [53]
			~ 1 mm						
	SEBS fiber	CNTs/rubber	2 mm	~ 160 μm	Rubber spray and wrapping	Prestrain releasing	Self-contact creases	Strain sensor	[60]
	PU	PVC	6–13 mm	50 μm	Adhesion	Dehydration	Gears		[52]
Hollow sphere	Actomyosin	Lipid monolayer	~ 8 μm	NA	Encapsulating	Contraction	Wrinkle		[209, 210]
	Latex balloon	Uniform GO film	2–15 cm	0.2–2.5 μm	Dip coating	Deflation	Cortex-like ridges	Actuator	[55]
		Bumpy GO film	2–15 cm	0.1–4.5 μm	Syringe dispensing	Deflation	Wrinkled papillae	Microdroplet manipulation	[54]
		CNTs	2–15 cm	~ 10^2^ nm	Adhesion	Deflation	Hierarchical ridges	Inflatable electronics	[64]
		MXene and CNT	2–15 cm	~ 1 μm	Adhesion	Deflation	Crumples	Flexible electronics	[62]
Cylindrical tube	Silicone tube	Parylene N	~ 1 mm	~ 2 μm	CVD	Stretching/releasing	High-aspect-ratio ridge		[181]
	Tubular balloon	Uniform GO film	0.8–5 cm	50–420 nm	Dip coating	Deflation	Hierarchical ridges	Wearable electronics	[61]
Micro-architecture	Bacteria	Graphene	~ 0.5 μm	2.5–3 nm	Direct deposition	Heat/vacuum	Wrinkled rod		[206]
	PEG-DA	PCP	10–60 μm	NA	Polymerization	Plasma	Wrinkled architectures	Cell culture	[185, 205]
	PDMS	Ag	~ 5 μm	20–200 nm	Electroless deposition	Deswelling	Winkled pillar	Pressure sensor	[82]

*PDMS* polydimethylsiloxane, *PS* polystyrene, *SEBS* styrene–(ethylene–butylene)–styrene, *CNTs* carbon nanotube sheets, *AgNWs* Ag nanowires, *PU* polyurethane, *PVC* polyvinyl chloride, *PAN* polyacrylonitrile, *BN* boron nitride, *GO* graphene oxide, *CVD* chemical vapor deposition, *PCP* partially cured polymer, *PEG-DA* poly(ethylene glycol) diacrylate, *MXene* 2D titanium carbide

## Applications

**Fig. 13:**
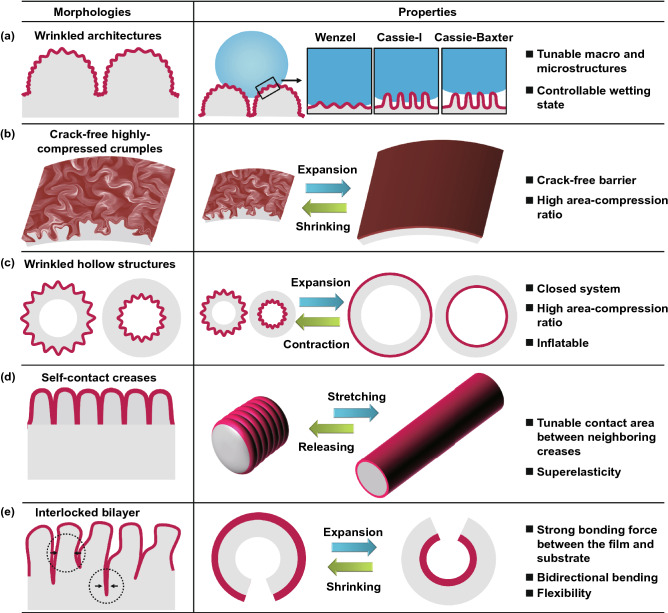
Superior properties of various wrinkled structures on curved substrates. **a** Wrinkling patterns can be generated on diverse microarchitectures with various geometries. **b** Crack-free highly compressed films can be obtained by simultaneous global deformations on curved substrates. **c** For closed hollow structures, wrinkle-to-smooth transition can be induced by reversible expansion and contraction. **d** Self-contact creases can be achieved on superelastic cylindrical fibers, where the contact area between neighboring creases can be tuned linearly by external strain. **e** Formation of highly convoluted interlocked structures that is capable of enhancing the bonding force between the film and the curved substrates

Controlled wrinkling on curved substrate opens new avenue for the fabrication of 2D patterns and 3D complex architectures on curved surfaces such as wrinkled papillae on spheres, crack-free highly compressed crumples on cylinders, periodic wrinkles on the inner surface of tubes, and self-contact creases on superelastic fibers (Fig. 13). These structures on curved substrates present a number of superior properties compared with wrinkling patterns on planar substrates. For example, multiscale wrinkling patterns can be obtained on the surfaces of 3D microarchitectures with various geometries, enabling controllable adhesion to liquids and cells [54, 185, 205] (Fig. 13a). Crack-free and highly compressed films can be obtained by simultaneous and isotropic shrinkage of the spherical substrate [55, 62] (Fig. 13b). By contrast, the formation of cracks in wrinkled stiff films is common on planar substrates due to Poisson effect [80]. Besides, a reversible wrinkled-to-smooth transition can be realized on the surface of hollow core–shell systems by simply varying the air amount or liquid volume in the cavity, enabling powerful control over the surface area [63, 64] (Fig. 13c). In addition, self-contact creases on superelastic fibers have superior stretchability (e.g., > 1000%) compared with that of wrinkled structures on planar substrates [53, 60, 78] (Fig. 13d). Moreover, normal displacement may trigger the formation of highly convoluted interlocked structures in curved core–shell systems, enhancing the non-chemical bonding force between the films and the substrates [55, 62] (Fig. 13e). By introducing more and more materials (e.g., CNTs, AgNWs, GO, MXene, and BN) into the wrinkling of curved core–shell systems, these superior properties derived from wrinkled structures on curved substrates open new avenue for emerging applications such as controllable adhesion [54, 205], biochemical protection [61, 200], electromagnetic shielding [62], friction [59], inflatable devices [64], wearable electronics [53, 60, 191], and strong actuators [55].

### Smart Wetting Surfaces

**Fig. 14:**
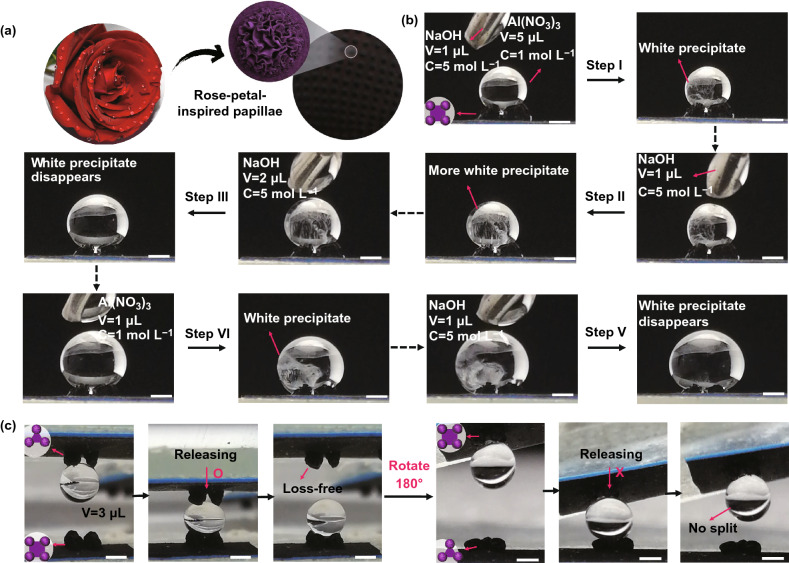
Superhydrophobic papillae arrays for microdroplet manipulation. **a** Rose-petal-inspired papillae array and SEM image of a papillae with fake colours. **b** Demonstration of the multi-step microreaction platform. **c** Snapshots show one-way transfer of a droplet between triangle array (*N* = 3) and square array (*N* = 4). All scale bars in the images are 1 mm [54]. Copyright 2019 Wiley

Wrinkled surfaces with tunable morphology are capable of controlling the wetting behavior and are crucial for applications such as self-cleaning [211, 212], microfluidics [213], and anti-icing [214]. For example, 1D wrinkle is demonstrated to be able to guide water spreading along wrinkle orientation leading to anisotropic wetting behavior [80, 211, 215–218]. 2D ridges/crumples with tunable aspect ratio enable programmable wetting transitions from Wenzel to Cassie state [219, 220]. Hierarchical structures enable superhydrophobicity for self-cleaning surfaces [48, 221–224]. Though the above-mentioned 1D and 2D wrinkling patterns on planar substrates have presented novel wetting properties, the fabrication of textured 3D architectures on curved substrates is critical for applications such as super-slippery surface [225, 226], microdroplet manipulation [54], and water harvest [227]. Hierarchical 3D architectures consisting of tunable microstructures enable powerful control over the adhesion to liquids [54]. For example, periodic hierarchical papillae enable the rose petal high adhesion to water under superhydrophobic state (*Petal Effect*) [18]. Similar structured papillae can be obtained by replication using natural rose petal as the template while template-free approach for achieving such microarchitectures is still difficult. Recently, Tan et al. [54] developed a simple template-free method for the fabrication of superhydrophobic papillae array with tunable adhesive force. The microdroplet on the papillae array exhibited a nearly perfect sphere and meanwhile the droplet kept hanging on the papillae even when the substrate was rotated 180°, presenting perfect petal effect. Possible applications of the superhydrophobic papillae in multi-step microreaction and programmable transfer of microdroplets were demonstrated (Fig. 14). A typical precipitation reaction of Al(NO_3_)_3_ and NaOH was taken as an example to demonstrate the superior performance of the papillae array for multi-step reactions (Fig. 14b). In addition, one-way transfer of a microdroplet between different papillae arrays can be realized by making a balance between the adhesive force of the papillae and the gravity force of the droplet (Fig. 14c).

### Cell Culture Interfaces

Surface morphology has important implications on regulating the proliferation and spreading of the cells [41, 70, 78, 228–233]. Wrinkling patterns with tunable sizes and orientations enable efficient control over the cell alignment. For example, Guvendiren and Burdick [41] observed that the stem cells on lamellar hydrogel pattern tend to spread along the wrinkles while the cells on hexagonal pattern tend to penetrate into the dimples with low spreading. Besides, the surfaces textured by 1D wrinkles or ridges have been demonstrated to be able to direct the cell alignment along the wrinkles or ridges compared with flat surfaces [70, 78]. Besides textured planar surface, controllable cell attachment on curved surfaces is crucial for investigating the mechanical behaviors of different cells and designing 3D cell culture interfaces. According to the contact guidance theory, cells tend to attach on flat surfaces instead of curved ones to minimize the deformation of their cytoskeleton [234, 235]. However, Li et al. [205] found that microwrinkles are capable of enhancing cell attachment on curved surfaces. For smooth posts on a flat substrate, almost all cells attached on the flat substrate and ignored the posts. For wrinkled posts on flat substrates, cell attachment was observed on 39.3% of the posts indicating that microwrinkles on the posts are capable of enhancing the cell attachment. Similar cell-attachment behaviors were observed on two distinctive round particles with smooth and wrinkled surfaces, respectively [31]. In addition, wrinkling patterns with tunable topography can also be used to regulate the friction on curved surfaces [59], which may provide some inspirations for designing functional surfaces for cell culture, soft robots, and drug delivery [236–238].

### Flexible Electronics

Controlled wrinkling is a simple and efficient approach for improving the flexibility of stiff conducting or semiconducting materials with poor deformability, which provides low-cost fabrication of flexible electronics and optoelectronics [33, 40, 87, 239–241]. For example, out-of-plane buckled silicon nanoribbons on soft substrates are able to accommodate large stretchability and are promising for stretchable and foldable electronic circuits [240, 242]. Besides buckled semiconducting films, the formation of wrinkling structures can also improve the stretchability of metal film remarkably. By attaching a patterned metal/elastomer bilayer to a prestretched mounting layer, Xu et al. [243] demonstrated that the formation of self-contact creases between two neighboring metal electrodes can be induced by releasing the prestrain, leading to the contact of two initially disconnected electrodes. This strain-driven contact and disconnect of the metal electrode can trigger large resistance change enabling sensitive strain-gated switches. Based on this strategy, multiple strain-gated logic transducer arrays have been developed [244, 245]. Recently, hierarchical rGO ridges have found some exciting applications such as stretchable pressure sensor [246, 247] and electrochemical capacitor with large areal specific capacitance [248].

**Fig. 15:**
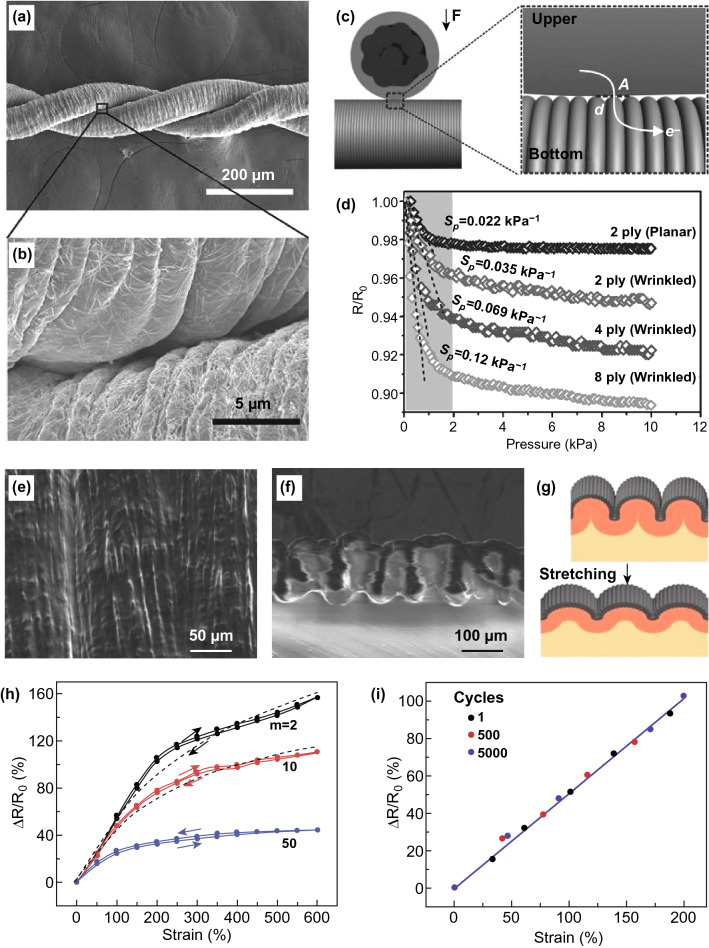
Self-contact creases on elastic fibers for flexible electronics. **a**–**d** Twisted flexible piezoresistive fiber: **a**, **b** SEM images of 2 ply creased fibers. **c** Schematic diagram of the sensing mechanism. **d** Relative resistance change with the variation of pressure [191]. Copyright 2016 Wiley. **e**–**i** A bi-sheath fiber for strain sensors: **e**, **f** SEM images of CNT creases on superelastic fibers, **g** schematic illustration of the separation of self-contact creases by external strain, **h** the resistance of the creased CNT shells decreases with reducing the contact area between neighboring CNT creases by applying strain, **i** stability evaluation of the bi-sheath fiber sensor [60]. Copyright 2017 Wiley

However, the maximum stretchability of the above-mentioned flexible electronics is below 150% and superelastic conductive wires and strain sensors for giant displacement require larger stretchability and better flexibility. With coating 1D materials (e.g., CNTs or AgNWs) on prestretched elastic fibers, superelastic conductive fibers can be obtained by releasing the prestrain [60, 190–192]. Liu et al. [60] fabricated hierarchically buckled sheath-core fibers with good electronic stability exhibiting a resistance change less than 5% for a 1000% stretch, which develops a new approach for superelastic electronics via strain engineering. By coating Ag NWs ink on a prestretched PU fiber, Wei et al. [191] fabricated multiscale creases on the fiber by releasing the prestrain (Fig. 15a, b). The obtained core–shell conductive fiber has high conductivity and good stretchability (400%). Besides, flexible piezoresistive fibers with high sensitivity (0.12 kPa^−1^) and fast response speed can be constructed by simply twisting multiple creased fibers into one bundle, since the resistance of the twisted fibers decreases by increasing the contact area between the upper and bottom creases and by applying a force *F* (Fig. 15c). Because of much more contact points on the creased fibers, the twisted creased fibers show higher sensitivity to the pressure than that of smooth fibers, and the sensitivity can be further improved by twisting more creased fibers into the system (Fig. 15d). By introducing a rubber layer between the prestretched SEBS fiber and the stiff CNT shells, Wang et al. [60] fabricated highly compressed self-contact creases on superelastic fibers after releasing the prestrain (Fig. 15e, f). The resistance between neighboring creases can be regulated linearly by applying external strain (Fig. 15g, h), and the core–shell fiber presents good stability even after 5000 stretching/releasing cycles (Fig. 15i). These superior properties make it a suitable candidate for resistive strain sensor with high linearity, fast responding, and excellent stability for detecting giant tensile and torsional displacements.

### Healthcare Materials

**Fig. 16:**
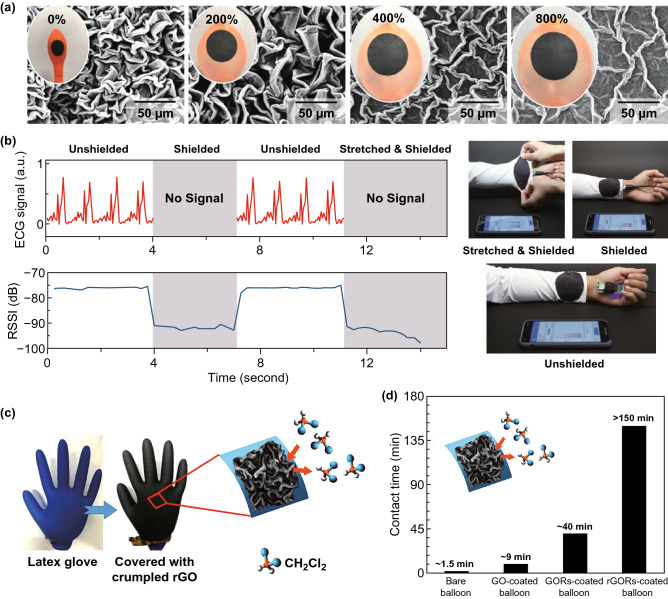
Highly compressed crumpled films for **a**, **b** stretchable electromagnetic shielding and **c**, **d** chemical barrier. **a** SEM images and digital photos of crumpled MXene-SWNT coating under various areal strains. **b** ECG signal and Bluetooth RSSI received by the smartphone with and without MXene-SWNT shield at its relaxed and stretching states [62]. Copyright 2019 Wiley. **c** Photos of a latex glove coated with hierarchical rGO crumples for stretchable chemical-protective uniforms. **d** Superior chemical-protective performance of rGO-coated glove, and no obvious damages are observed even after immersing into dichloromethane over 2.5 h [61]. Copyright 2019 American Chemical Society

In many situations, stretchable materials with excellent chemical barrier and electromagnetic shielding property are critical for protecting the human body exposed to toxic chemicals and strong electromagnetic (EM) fields [249, 250]. Among the alternative materials, 2D graphene and MXene have been demonstrated to be promising candidates for chemical and electromagnetic shielding [251–255]. However, poor stretchability and the formation of defects may reduce their protective performance in practical applications. By transforming 2D materials into highly compressed and crack-free wrinkled microstructures, the flexibility of the film can be improved remarkably [55]. For example, stretchable highly compressed rGO films, inflatable hierarchically buckled CNTs, and crumpled MXene coatings have been fabricated by the deflation of latex balloons [61, 62, 64, 200]. These crumpled films have been demonstrated to be capable of protecting human body from chemical corrosion [61], electromagnetic radiation [62], and some of them can be used as inflatable devices including inflatable tumor ablation and inflatable antenna [64]. By coating MXene-SWNT films on inflated balloons, Li et al. [62] realized reversible crumpling of the MXene-SWNT coating by increasing the areal strain from 0 to 800%, and no damages were observed during the deformation process (Fig. 16a). The crumpled MXene-SWNT/latex bilayer was capable of attenuating electromagnetic absorption by the human body under both relaxed and stretching state (Fig. 16b). By using similar technical approach, Song et al. [61] demonstrated that the highly compressed rGO films have enhanced chemical stability compared with that of smooth ones (Fig. 16c, d). By coating a highly crumpled rGO film onto a latex balloon, the balloon remains inflatable even after contacting with dichloromethane for more than 2.5 h that is about 100 times longer than that for bare balloon (Fig. 16d). Recently, Liu et al*.* [64] fabricated several inflatable devices such as tumor ablation, volumetric sensor, and inflatable antenna by using latex balloons covered with highly convoluted CNT films. The obtained inflatable devices exhibited good stability under repeated inflation/deflation cycles.

The attachments of thrombus, virus, and pathogenic bacterial on artificial tissues are harmful for human health and how to avoid such depositions is a big challenge for designing and fabricating biocompatible self-cleaning surfaces. Wrinkled surfaces modified with functional groups or Ag nanoparticles have been demonstrated to be able to prevent thrombus formation and bacteria attachment [256–258]. For example, Yuan et al. [256] fabricated a slippery surface by infusing fluorocarbon liquid into wrinkling structures. The obtained slippery surface is capable of preventing the platelet adhesion and suppressing thrombus formation. Besides, Efimenko et al*.* [259] demonstrated that hierarchically wrinkled coatings could be a potential candidate for marine antifouling. Recently, Pocivavsek et al. [63] fabricated an anti-thrombotic tube with wrinkling morphology on its inner surface. Similar to the shrinkage and expansion of arteries, thrombotic deposition on the wrinkled surfaces can be removed efficiently via a reversible smooth-to-wrinkle transition induced by pressure.

### Actuators

**Fig. 17:**
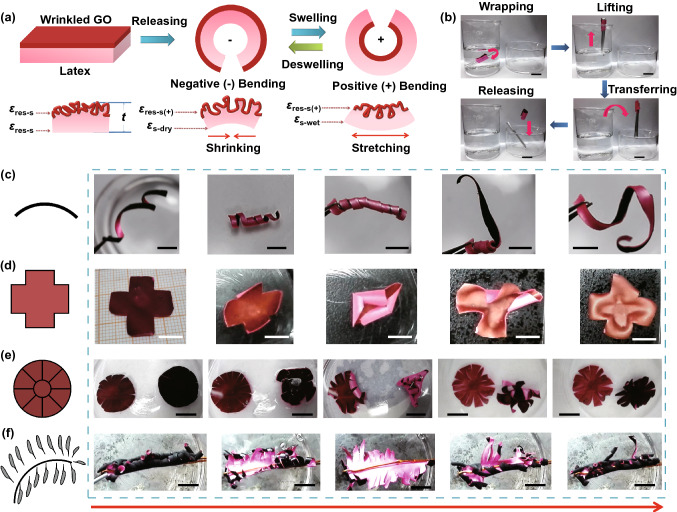
Typical application cases of the wrinkled GO/latex bilayer actuators. **a** Schematic illustration of the mechanical self-assembly of the bilayer actuators. When the wrinkled GO/latex bilayer sphere is cut into slices, the obtained bilayer sheets bend spontaneously to partially release the residual stress stored in the system, which can also bend backward in response to some organic solvents, and could recover to its initial bending state after drying in air. **b** A bilayer actuator used as a mechanical hand grasping cylindrical Al foil in *n*-hexane. **c**–**f** Digital images of the bending and recover process of **c** a worm-like swimmer, **d** a smart package, **e** two bionic flowers, and **f** a bionic mimosa. All scale bars in the images are 1 cm [55]. Copyright 2017 American Chemical Society

Many plants are capable of responding to external stimuli by changing their shapes [260]. For example, the mimosa is sensitive to mechanical stimuli and its leaves quickly close together once being touched [261]. Stimuli-responsive behaviors of these plants without a nervous system have fascinated researchers to develop bionic actuators that respond quickly to the environmental stimuli [262]. For most asymmetric actuators, weak non-chemical bonding force, low response speed, and small deformation limit their applications [263]. The formation of wrinkled microstructures has been demonstrated to be able to improve the deformation and response speed of asymmetric actuators [264, 265]. For example, Li et al. [264] reported a wrinkled hydroresponsive actuator with anisotropy, gradient, and instant response. Compared with flat actuator, the actuator with wrinkled hydroresponsive layer presents much faster response and larger bending angle. Qiu et al. [265] demonstrated that a sole GO film composed of a wrinkled layer and a smooth layer is also capable of providing moisture-actuation due to hygroscopicity of GO. Besides, highly compressed conductive wrinkles, crumples, and ridges were also demonstrated to be excellent electrodes for enhancing the bending and stretchability of dielectric elastomer actuator (DEA) [220, 266–268]. The formation of interlocked structures in curved bilayers enables stronger interaction force between the substrates and the films, enabling the wrinkled curved bilayers suitable candidates for asymmetric actuators with large deformation and excellent stability [53, 55]. By combining excellent flexibility of highly convoluted GO films and the swelling of latex substrates, Tan et al*.* [55] achieved a solvent-responsive asymmetric actuator with fast responding speed, bidirectional bending, and large deformation (Fig. 17). Impressively, the actuator presented an ultra-large curvature change up to 2.75 mm^−1^, which can be used as a powerful gripper to grasp and transfer smooth objects in toxic solvents (Fig. 17b). Besides, multiple types of biomimetic actuators can be fabricated by cutting the highly convoluted GO/latex bilayers into 1D strip, 2D sheet, and 3D complex architecture (Fig. 17c–f). Controlled wrinkling on curved substrates makes it possible to design strong actuators that are capable of changing their shapes among various dimensions, which may inspire scientists to develop bionic self-adaptive systems with multifunction [269].

**Fig. 18:**
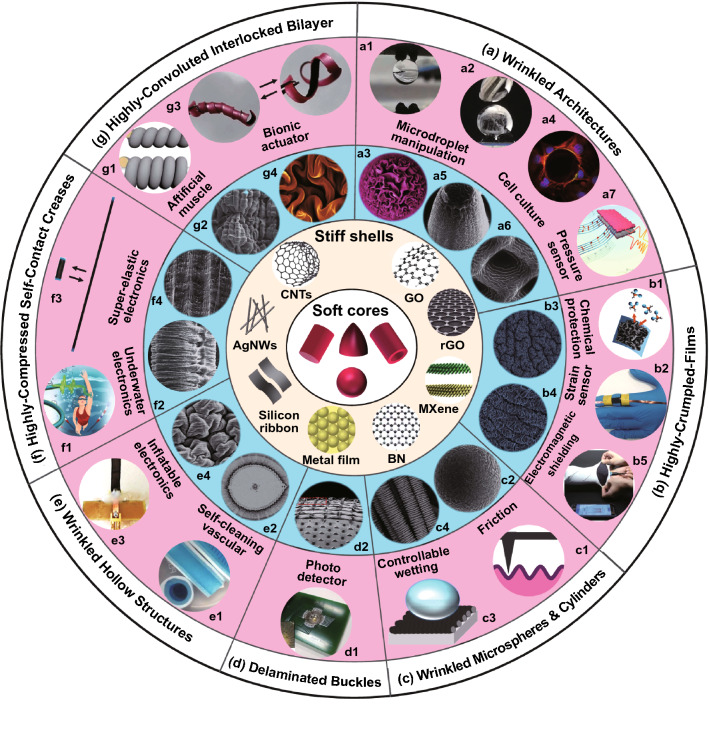
Applications of diverse wrinkling structures on curved substrates. **a** Wrinkled architectures for controllable adhesion. **a1-a3** Flower-like papillae for microdroplet manipulation [54]. Copyright 2019 Wiley. **a4-a6** Patterned microarchitectures for cell attachment [185, 205]. **a4** Adapted with permission from Ref. [185]. Copyright 2016 Springer Nature. **a5**,**a6** Adapted with permission from Ref. [205]. Copyright 2015 Wiley. **a7** Textured micropillars for pressure sensor [82]. Copyright 2016 American Chemical Society. **b** Highly compressed crumpled films for chemical protection and electromagnetic shielding. **b1-b4** Hierarchical rGO crumples for chemical protection and strain sensor [61]. Copyright 2019 American Chemical Society. **b5** Flexible MXene crumples for electromagnetic shielding [62]. Copyright 2019 Wiley. **c** Wrinkled microspheres and microcylinders for friction and controllable wetting. **c1**,**c2** Wrinkled metal-coated PDMS microspheres for friction [59]. Copyright 2019 Wiley. **c3**,**c4** Ring-patterned fibers for controllable wetting [58]. Copyright 2019 Elsevier. **d** Delaminated buckles for photodetector [56]. **d1**,**d2** Adapted with permission from Ref. [56]. Copyright 2010 Wiley. **e** Wrinkled hollow structures for inflatable devices. **e1**,**e2** Self-cleaning vascular [63]. Copyright 2019 Wiley. **e3**,**e4** Inflatable antenna [64]. Copyright 2019 American Chemical Society. **f** Self-contact creases for superelastic electronics. **f1** Underwater wearable electronics [190]. Copyright 2019 Wiley. **f2** Piezoresistive fiber [191]. Copyright 2016 Wiley. **f3**,**f4** Superelastic strain sensor [60]. Copyright 2017 Wiley. **g** Highly convoluted interlocked bilayers for strong actuators. **g1**,**g2** Hierarchically buckled sheath-core fibers for artificial muscle [53]. Copyright 2015 The American Association for the Advancement of Science. **g3**,**g4** Highly convoluted GO patterns for bionic actuator [55]. Copyright 2017 American Chemical Society

According to the geometries of soft cores, materials of stiff shells, and typical wrinkling morphologies, the applications of diverse wrinkled structures on curved substrates are summarized in Fig. 18. Besides the above-mentioned applications, wrinkling patterns on curved substrates have some other potential applications such as hemispherical electronic eye camera [270, 271], microlens arrays [57, 272], and aerodynamic drag control [273].

## Conclusions and Outlook

In this review, we provide an overview of wrinkling mechanisms, fabrication methods, and applications of bio-inspired wrinkling patterns on curved substrates. In the past decade, much progress has been made in both fabrications and applications of wrinkled structures in curved core–shell systems. More and more building blocks such as metal coatings, low-dimensional materials, and semiconductors were used to fabricate functional wrinkled structures on curved substrates, and meanwhile a number of novel topographies such as wrinkled architectures, highly compressed self-contact creases, highly convoluted interlocked structures, large-area crack-free crumpled films, and wrinkled hollow structures have been achieved by controlling parameters like the core–shell modulus ratio, substrates curvature, shell thickness, and deformation models. These wrinkled morphologies with special properties remarkably extend the applications of structured curved surfaces to superelastic electronics, bionic actuators, controllable adhesion, chemical protection, artificial vascular, and inflatable devices [53–64].

However, there is still a long way to go for understanding the wrinkling of curved biological surfaces and biofabricating wrinkled structures with multiple functionalities: (i) the wrinkling mechanism of hierarchically folded structures on curved substrates under ultrahigh mismatched strains is still unclear, which is significant for understanding the formation mechanism of complicated multiscale structures observed on biological surfaces (e.g., folds, villi, and microvilli of the small intestine). (ii) Surface instability in soft matter is a simple approach to mimic the mechanical wrinkling of human tissues with growth, but the core and shell materials used in previous studies are far different from the actual physiological environment. More types of bioactive materials should be introduced into the core–shell systems for the biofabrication of wrinkled patterns on soft tissues [274]. (iii) Program-controlled and on-demand fabrication of wrinkling structures on curved substrates are still a challenge since precise regulation of orientations and feature sizes of wrinkling patterns on diverse architectures remains difficult. More deformation models are needed to control the surface topography of stiff shells on soft cores.

In spite of these challenges, we believe that mechanical self-assembly based on controlled wrinkling on curved substrates will become one of the most significant 3D micro/nanofabrication technologies in future. By introducing more and more functional building blocks such as low-dimensional materials, bioactive materials, and information storage modules in the wrinkling of curved core–shell systems, these emerging techniques may find applications in the biofabrication of artificial organs, four-dimensional (4D) printing, and 3D assembly of micro/nanochips for artificial intelligence.
